# Decoding autonomy in digital work: how direct, indirect, and moderated effects shape health and burnout of social workers

**DOI:** 10.1186/s12889-026-27041-9

**Published:** 2026-03-20

**Authors:** Eva Gnugesser, Marlies Jöllenbeck, Wiebke Schlenger, Elke Ochsmann

**Affiliations:** 1 Institute of Occupational Medicine and Public Health, Medical Faculty, Saarland University, Campus Homburg, Kirrberger Str. 100, Homburg, 66424 Germany; 2 Employer’s Liability Insurance Association for Health Services and Welfare Care (BGW), Hamburg, 22089 Germany; 3 Institute of Occupational Medicine, Prevention and Workplace Health Management, Medical Faculty, University of Luebeck, Luebeck, 23562 Germany

**Keywords:** Occupational health, Counselling, Job demands–resources, Work organisation, Digitalisation

## Abstract

**Background:**

The increasing use of digital technologies is reshaping working conditions and employee well-being. Although autonomy is often described as an important resource for digital work-related health, findings remain inconsistent, particularly in health and social work contexts. In these settings, digital work practices are becoming increasingly common, with counselling now frequently conducted by phone, email or video via company-specific online counselling platforms rather than traditional face-to-face methods. This study examines whether and how autonomy is related to health in these digital workplaces through direct, indirect, and moderated associations.

**Methods:**

Social counsellors from a large German social welfare organisation, with varying frequencies of online counselling, completed a cross-sectional, web-based questionnaire assessing autonomy, job demands, resources, work-life balance (WLB), and health outcomes using validated scales. Data analyses included mediation and moderation models to examine indirect and interactional associations.

**Results:**

Autonomy was significantly related to digital counselling frequency, with higher levels of autonomy observed among counsellors who more frequently used digital work practices. Although autonomy was positively correlated with work-related health, the association was not direct. Instead, autonomy’s impact emerged through interactions with specific job demands and resources, significantly buffering negative associations of work interruptions and the work environment with well-being. Moreover, intersectional WLB was identified as a key mediator in the indirect associations between autonomy and health, as well as between autonomy and burnout.

**Conclusion:**

Enhancing work autonomy can be beneficial in digital social work, as it can promote health and reduce burnout. However, these benefits are context-dependent, underscoring the complexity of digital work settings and the need for careful contextual analysis before implementing workplace interventions. Future research should further explore digital environments prior to enhancing work autonomy, particularly among high-strain employees such as social workers.

**Supplementary Information:**

The online version contains supplementary material available at 10.1186/s12889-026-27041-9.

## Introduction

The health and social care sector (HeSCare; encompassing health care, residential care, and social work) is a major occupational field in the EU, employing approximately five million people in social work alone. By providing counselling services on issues such as addiction or pregnancy for vulnerable groups, as well as guidance on nursing activities in domestic care, these occupations are of high societal importance [1].However, the combination of structural issues such as low pay and effort reward imbalance with sector-specific demands including high workload and emotional strain places HeSCare workers at elevated health risk that threatens the stability of this essential workforce [1–3].

While complex, the occupational risk profile reflects a coherent pattern of work-related strain where professional demands regularly outpace available resources (“job strain”) [1]. According to the EU-OSHA, over 50% of workers experience this kind of 'job strain' with demands not limited to high work intensity but including interpersonal demands such as having to deal with difficult clients – with 18% of workers reporting this issue the highest proportion across all occupations [1]. When these psychological and social demands occur simultaneously without sufficient resources to mitigate them, they might result in significant somatic and mental health consequences [4]. Therefore, it is not surprising that over one-third report harmful work-related health effects, including musculoskeletal pain, headaches or eyestrain, anxiety, and burnout [1].

Recently, the COVID-19 pandemic and its acceleration of the digital transformation from analogue to digital counselling via telephone or video [5], have added a new layer of complexity to the existing strain profile [6]. In Germany, video counselling alone increased from 1.5% to 7.7%, and telephone counselling from 24.7% to 28.0%, while face-to-face contacts dropped from 35.5% to 16.7% [7]. These abrupt structural changes in service delivery have been accompanied by an increase in stress related symptoms in over one-third of social workers [3], driven largely by blurred work-life boundaries, increased workloads, and staff shortages [1]. Additionally, persistent technical and infrastructural challenges, such as inadequate IT equipment, remain major stressors, particularly in underfunded social care and nursing settings where such tools are now indispensable for daily operations [2].

Conversely, digital tools have also displayed improvements to employees’ flexibility to work whenever and wherever they want and hence seem to support better work-life balance (WLB), defined here as an individual’s subjective attitude toward their life situation in regard to combining different roles in private and work life [2, 8, 9]. More specifically, WLB reflects the balance between one's personal aspirations and the actual reality of their various roles and goals. Better WLB has been associated with various beneficial health outcomes including lower mental health symptoms via enhancing psychosocial resources and job satisfaction [10, 11]. As a result, burnout, a multidimensional construct of physical, emotional, and mental exhaustion (e.g., resulting from chronic workplace stress) was reported to decrease with higher WLB [10–14]. Frequent engagement with digital counselling has also been directly linked to reduced cognitive and sleep disturbances, suggesting adaptation to new digital demands after an initial adjustment period [6]. Ultimately, the itemised physical, mental and psychosomatic effects can sum up to alter the individual self-perceived health status. Self-rated general health serves as an overall assessment of a person’s general state of health based on subjective perception, integrating physical, social and emotional functioning and disease-relevant aspects and symptoms [15]. A more negative self-assessment of general health correlates with more frequent health complaints and chronic diseases and is an important predictor of functional and cognitive impairments, utilisation of health services and mortality.

Given the already above-average strain levels of HeSCare workers, enhancing benefits and limiting detrimental effects is critical during the digital transformation. Workplace autonomy has emerged as a potential job resource that could play this role. Defined as the degree of control an employee exercises over their work, work autonomy is characterised by three distinct but related concepts: planning autonomy (scheduling and timing), method autonomy (procedures and execution), and decision-making autonomy (influence over workplace choices) [16]. Previously, workplace autonomy has been linked to improved health, reduced burnout, and greater well-being in the HeSCare sector [17, 18].

While autonomy has been widely examined in traditional in-person settings, its function in the rapidly evolving digital social work environment and its role in positively influencing the digital transformation process remains less clear. It is currently unknown whether and through which mechanisms autonomy improves or worsens employee well-being in digital social care—a critical concern for a profession already facing workforce shortages. Because the pandemic-driven digital transition was so abrupt, many organisations may have lacked the evidence-based frameworks needed to manage these dynamics, potentially leading to the precautionary retraction of autonomy-enhancing measures and inefficient allocation of already limited resources to less effective workplace measures.

Rather than treating autonomy as a uniform resource, this study extends existing research by investigating and comparing the direct, indirect, and interactional associations of autonomy with employee wellbeing as a multidimensional construct within the Job Demands–Resources (JD-R) framework to better understand its complex mechanisms in the digital social care landscape. By focusing on social counsellors in a large German welfare organisation that is increasingly implementing online and blended counselling strategies, this study is situated directly within the context of digital transformation. While the scope is specific, there are substantial overlaps with other health professionals considering social care’s wide occupational profile ranging from addiction to pregnancy counselling and disability support.

### Theory and hypotheses

Occupational demands and resources, including autonomy, can vary by job type, role, and organisational context. Professions that emphasise achievement, creativity, and independence tend to offer higher autonomy, supported by opportunities for personal initiative and competence [19]. Hierarchical status is also relevant: managers or self-employed individuals often experience more autonomy than blue-collar workers [20]. In social care, autonomy is often context-dependent, allowing freedom in scheduling and case management while facing constraints from legal regulations and supervision [20, 21]. Digitalisation further complicates these conditions: On the one hand, digital tools can foster autonomy by decoupling work from fixed face to face methods, fixed locations or fixed consultation hours, allowing counsellors to choose where, when and how they engage with clients [22–24]. On the other hand, the new technologies can facilitate a form of restriction, where work processes are standardized through mandatory software or digital tool use or monitored through digital surveillance and accompanied with ambitious organisational goals (e. g. reaction time to a digital query) [22–24]. In this complex web of associations, we argue that autonomy may change with increasing digital counselling use in the knowledge-based counselling sector. As the interplay of positive and negative effects of digitalisation on autonomy remains unclear among counsellors, the direction of change it is still an open question. We therefore formulate two competing hypotheses:

- H1a : Higher levels of digital counselling use among social workers are associated with higher levels of autonomy.
- H1b: Higher levels of digital counselling use among social workers are associated with lower levels of autonomy.

According to the JD-R framework, high (digital or transformational) demands and low (workplace) resources each undermine well-being independently (additive effect), whereas sufficient resources can buffer the negative effects of high demands (interaction effect) and moderate the relationship between working conditions and health outcomes [25, 26]. While additive effects are more consistently supported in the literature, interaction effects appear particularly relevant in HeSCare settings [25, 27]. In line with its role as an occupational resource, autonomy may also exhibit distinct additive (independent) and interactional effects.

Autonomy – a major occupational resource according to the JD-R model—has been shown to interact with employee well-being and workplace characteristics in various ways [17, 18]. Despite the potential benefits of autonomous working conditions, the interplay between autonomy and other psychosocial workplace factors (e.g. those recommended by the ‘Joint German Occupational Safety and Health Strategy’ (GDA) [28]) and how these pathways shape well-being remains underexplored. Importantly, it is not sufficient to rely solely on theoretical or experimental approaches. Studying autonomy within real workplace contexts, where multiple factors interact, is essential. These factors include both demands (e.g. workload, emotional burden, interruptions, collaboration requirements) and resources (e.g. social support, task variety), which together influence employees’ well-being and the effectiveness of autonomy-supportive interventions.

WLB is another key determinant of employee health, especially in digitally connected work environments [29]. While the JD-R model typically focuses on occupational factors, WLB encompasses both work and personal-life demands and resources. In digitalised settings, the boundaries between work and home life are increasingly blurred, making it essential to consider WLB as a potential factor in occupational health research [30–32]. Drawing on these theoretical assumptions, we propose the following hypotheses:

- H2a: Higher levels of overall job autonomy are positively associated with employee health, independent of job demands (information burden, workload, emotional burden, interruptions, need for collaboration), job resources (social support, opportunities for collaboration, task variety), and WLB.
- H2b: Higher levels of overall job autonomy are negatively associated with burnout, independent of job demands (information burden, workload, emotional burden, interruptions, need for collaboration), job resources (social support, opportunities for collaboration, task variety), and WLB.

However, autonomy may not only serve as an independent resource, but may also actively shape other workplace characteristics (mediation) and their relationships (moderation) with employee well-being [18, 33, 34]. A growing body of research suggests that autonomy enables employees to engage in proactive work behaviours, such as job crafting—actively modifying job demands and resources to better align with their personal needs, skills, and values – thereby enhancing well-being [18]. In this context, autonomy also plays a pivotal role in shaping WLB [35]. High planning autonomy provides employees with greater control over when, where, and how they perform their work, facilitating effective integration or segmentation between life domains according to individual needs. For instance, employees with higher planning autonomy may adjust their working hours to accommodate family responsibilities, a practice that has been linked to improved WLB and, in turn, higher job satisfaction [36]. In line with these considerations, we propose the following hypothesis:

- H3: Autonomy exerts indirect associations with employee well-being via job demands, job resources, and WLB, specifically:
  - H3a: improved health outcomes and
  - H3b: reduced burnout symptoms.

Beyond its direct and indirect effects, autonomy may also function as a moderator within the workplace context. According to the original JD-R model, job resources such as autonomy can buffer the negative relationship between demands and well-being [25, 26], for example, by providing coping strategies to counter the negative effects of job demands [4, 37]. Based on appraisal theory, the detrimental effects of job demands on employee well-being are heightened when they are perceived as hindrances rather than challenges [38]. Autonomy has the potential to change these perceptions of demands, as shown in studies on work interruptions. In such contexts, interruptions were more likely to be appraised as manageable challenges under high-autonomy conditions, whereas they were more often regarded as hindrances under low-autonomy conditions [39]. These theoretical assumptions lead to our fourth hypothesis:

- H4a: Autonomy moderates the relationship between job demands, job resources, and WLB and health outcomes.
- H4b: Autonomy moderates the relationship between job demands, job resources, and WLB and burnout.

The findings aim to inform HeSCare employers and employees, particularly in the field of social care, but also in related occupations such as nursing, as well as the HeSCare community more broadly. They also seek to clarify whether workplace interventions designed to enhance digital autonomy can serve as potential facilitators of employee well-being, and, if so, which positive health effects can realistically be expected from such interventions.

## Methods

### Study design, setting and sample

This cross-sectional study analysed data from a national online survey conducted between April and June 2022 among employees of a large German social welfare organisation with HeSCare centres across Germany, which also operates internationally. The survey, part of a broader project on digitalisation in social care, has been described in detail by Gnugesser et al. [6] Organisational participants were recruited through convenience sampling. A web-based questionnaire was distributed to digital counsellors with varying degrees of digital work via an intra-organisational mailing list. No further eligibility criteria were applied. The organisation’s list of digital HeSCare workers included approximately 5,000 employees, yielding a response rate of approximately 20% (n = 1,049). Reminder emails were issued every two weeks to maximise participation. Data were collected using the SoSci Survey software [40]. Ethical approval was granted by the University of Lübeck Ethics Committee (AZ 21–388, Date: 15.10.2021). The study was conducted in compliance with the principles of the Declaration of Helsinki. Electronic informed consent was obtained prior to participation, and only anonymised data were analysed in this study.

### Instruments with validity and reliability

The survey was developed for a larger project on digitalisation in social care (Gnugesser et al [6].). It combines validated scales with newly created job-exposure items tailored to sector-specific aspects of digital transformation. Standardised scales measured psychological and organisational constructs (job demands and resources, autonomy, work–life balance, burnout), while study-specific items captured sociodemographic (age, gender) and work-related variables (counselling field, experience with digital formats, extent of online counselling). These customised items were developed with stakeholders to ensure contextual relevance and cover formats such as video, email, chat, and specialised software [5]. Detailed descriptions of all validated scales and response formats appear in the variable sections. Because the questionnaire is part of an ongoing research programme with further analyses planned, the full instrument is not presented; only the subsection relevant to this study is provided in Additional file 2, including the original German version and an English translation for transparency. The English translation was not used in data collection.

Age and self-reported gender were included as potential confounders in the models, as age and gender differences are well-known, for example, in relation to burnout risk [41]. For latent constructs consisting of several questionnaire items, composite scores were calculated by averaging the corresponding Likert-scale items in accordance with the validated questionnaires and the proposed factor structure. Details regarding the specific item composition of each variable are provided in the original publications of the validated instruments [9, 20, 42, 43]. This procedure yielded quasi-metric variables on the original 1–5 scale (for autonomy, demands, and resources) and the 1–6 scale (for WLB), allowing them to be treated as continuous while maintaining interpretability. The composite scores were subsequently used in the main regression analyses.

To assess robustness, a confirmatory factor analysis (CFA) was conducted to examine whether the proposed factor structure for the Likert-scale items measuring autonomy, demands, resources, and WLB was supported by the data [44]. Model fit was evaluated using commonly reported indices [45]: the comparative fit index (CFI), the Tucker–Lewis index (TLI), the root mean square error of approximation (RMSEA), and the standardised root mean square residual (SRMR). CFI and TLI values of 0.90 or higher, RMSEA values of 0.08 or lower, and SRMR values below 0.08 are generally considered indicative of an acceptable model fit [45]. It should be noted, however, that RMSEA values can be misleading for models with few degrees of freedom [46]. In line with the recommendations of Kenny et al., model fit was therefore evaluated by considering all fit indices collectively. [46] Analyses were performed using Jamovi and the ‘lavaan’ package [47, 48], with the WLSMV (weighted least squares means and variance adjusted) estimator employed to account for the ordinal nature of the Likert-scale items [49]. Overall, the CFA models demonstrated satisfactory fit according to widely accepted criteria, although minor deviations were observed in RMSEA, likely due to the limited degrees of freedom. These CFA results were used solely for robustness purposes and were not included in the main regression analyses. A detailed overview of the model fit parameters is provided in the variable-specific description sections and in the Appendix (Additional file 1).

#### Autonomy

Work-related autonomy was assessed using the German version of the ‘Work Design Questionnaire’ (WDQ), which covers the three autonomy dimensions of planning, methods, and decision making [20]. Each dimension was measured on a 5-point Likert scale and later combined into a composite score ranging from 1(lowest) to 5 (highest perceived autonomy). The WDQ has demonstrated good internal consistency across the different subsets of autonomy (planning (Cronbach’s α = 0.85), methods (α = 0.88), decision making (α = 0.85)) in the original study among German employees. These values were confirmed in the present study for the subscales (planning (α = 0.82), methods (α = 0.84), decision making (α = 0.87)) and the combined score (α = 0.90). Drawing on previous theoretical and empirical work, we modelled autonomy as a second-order factor in the CFA, following an approach used in prior research on teachers’ perceptions of autonomy-supportive behaviour in physical education [50]. This approach captures an overarching construct that integrates several conceptually related yet distinct subdimensions, providing a more parsimonious and interpretable representation of the data [51]. The three subdomains (planning, methods, decision making) represent distinct yet related aspects of the broader construct of autonomy [16]. Each subdomain loaded strongly on the higher-order factor (standardised factor loadings λ = 0.76–0.94), and the model demonstrated acceptable fit (CFI = 0.99, TLI = 0.99, RMSEA = 0.07 (*p* < 0.001), SRMR = 0.04). This approach provides a concise and efficient representation of the overall construct while preserving the meaningful subdimensions. To account for the respective subdimensions, separate regression analyses are presented in the Appendix (Additional file 1).

#### Working conditions

Following the guidelines of the GDA [28], a systematic risk assessment was conducted using the ‘Short Questionnaire for Workplace Analysis’ (KFZA), a validated German instrument designed to capture various psychosocial job demands and resources in the workforce [42]. Internal consistency was heterogeneous across the individual demands and resources (α = 0.61–0.87), but in line with reference values (α = 0.60–0.76), which were theoretically justified by the authors of the original study [42]. The assessed work demands included quantitative workload, work interruptions, environmental burden, and information burden. Collaboration was operationalised as both a demand (“My job demands collaboration with colleagues or clients”) and a resource (“I can talk with colleagues about work-related and private matters” and “I regularly receive feedback from supervisors and colleagues”). Social support and variety were also included as resource variables drawn from the KFZA [42]. Information burden was reverse-worded but scored in the same direction to maintain interpretive consistency. Emotional burden, another job demand, was assessed using selected items from the German version of the ‘Copenhagen Psychosocial Questionnaire’ (COPSOQ), which is validated for use in occupational health research [43]. All factors were measured using Likert-scaled items ranging from 1(“not at all”) to 5 (“completely agree”), with higher scores indicating higher levels of the respective demand or resource. Therefore, the resulting quasi-metric composite variables ranged from 1 (lowest) to 5 (highest). Single-factor models for demands and resources were tested using CFA, based on the KFZA factor structure (see Additional file 1) [42]. The demands model showed acceptable fit (CFI = 0.97, TLI = 0.96, RMSEA = 0.09 (*p*< 0.001), SRMR = 0.07), although some limitations emerged, particularly for the variable “interruption”, which also displayed low internal consistency in the original KFZA validation study [42]. This item reflected two dimensions: interruptions from external contacts and from insufficient work materials. However, combining these aspects into a single latent factor was justified by the original authors, as it represents the cumulative effects of different interruption types as “micro stressors” [42]. The resources model showed good fit (CFI = 0.99, TLI = 0.99, RMSEA = 0.06 (*p* = 0.25), SRMR = 0.04), supporting the use of these items as valid indicators of the latent resource constructs (see Additional file 1).

*WLB*. Although not a traditional work condition, WLB was included as a covariate due to its well-established links with employee well-being [52]. It was measured using the validated ‘Trierer Scale to Measure Work-Life Balance’ (TKS), which consists of five items with high internal consistency in the original (α = 0.88–0.95) and our study sample (α = 0.89) [9]. Items were rated on a 6-point scale from 1 (“not satisfied with WLB”) to 6 (“very satisfied with WLB”) [9]. The CFA indicated an acceptable model fit (CFI = 0.99, TLI = 0.99, RMSEA = 0.09 (*p* = 0.007), SRMR = 0.03).

#### Health and burnout

Two key health-related outcomes were examined: general health and burnout. Current self-perceived general health was assessed using a single item rated on a 10-point numerical scale ranging from 0 (“worst perceived health”) to 10 (“best perceived health”). Burnout symptoms over the past three months were measured using the German version of the COPSOQ burnout scale, rated on a scale from 0 to 100, with higher scores indicating greater burnout symptoms [43]. The scale has demonstrated good internal consistency in previous research (α = 0.84), which was confirmed in our study (α = 0.86).

### Data analysis

Missing data were handled via listwise deletion based on the included variables [53]. As the data deviated from normality, as indicated by histograms and Q-Q plots, Spearman’s rank correlation coefficients (rho) were calculated for bivariate associations. Differences in autonomy levels by online counselling frequency were examined using a Kruskal–Wallis test (see Additional file 1). Associations between autonomy (total and subdimensions) and the two primary outcomes—general health and burnout—were examined using separate multiple linear regression models, adjusting for age, gender, job demands (six variables), job resources (three variables), and WLB. Hierarchical regression analyses were conducted, with predictors entered in theory-driven blocks. The models were estimated in five steps: in the first step, the control variables age, gender, and online counselling frequency were entered, and afterwards reported as adjustment variables. In the second step, the primary independent variable of interest, autonomy, was added to assess its significance beyond the control variables. In subsequent steps, additional predictor blocks were entered sequentially: job demands (third step), job resources (fourth step), and WLB (fifth step). This stepwise entry procedure allowed us to evaluate changes in explained variance and the stability of regression coefficients across models. Age, gender, and online counselling frequency were measured and included as categorical covariates, while all other predictors were treated as continuous variables. Mediation and moderation analyses were conducted using the PROCESS macro (v4.2) for SPSS [54]. Bootstrapping procedures (10,000 samples) were applied to generate bias-corrected 95% confidence intervals, and robust standard errors (HC3) were used to account for heteroscedasticity. Mediation models tested the indirect associations of autonomy with the outcomes through individual job demands, resources, and WLB. Moderation models included linear interaction terms between autonomy and each respective demand, resource, and WLB. Each interaction was tested in a separate regression model rather than all interactions being entered simultaneously. Variables included in the interaction terms were standardised prior to computation. When significant interactions were identified relative to the base model, simple slope analyses were conducted and visualised using interaction plots. Model assumptions, including linearity, multicollinearity, homoscedasticity, and normality of residuals, were evaluated and met based on Shapiro–Wilk tests, scatterplots, and correlation diagnostics. Model fit was evaluated using adjusted R [2] [55]. All statistical analyses were performed using SPSS (Version 29), with a significance threshold of *p* < 0.05. Interaction plots were created using R software (v4.4.2).

## Results

### Characteristics of the sample

In total, *N* = 1,049 employees participated in the survey. Some variables showed notable missing data, with the highest proportion observed for social support (n = 48, 4.6%), resulting in n = 906 included datasets after listwise deletion. The sample was predominantly female (76.6%), with most participants aged between 36–50 years (35.3%) or 51–65 years (47.0%). Nearly half of the participants reported up to two years of experience in online counselling, likely reflecting the increased uptake during the COVID-19 pandemic. The most common counselling fields were “family and parenting” (20.2%), “pregnancy” (14.4%), and “addiction” (12.8%). Approximately 65.3% of counsellors reported that 10% or less of their monthly work activity was spent on digital work, reinforcing the view that digitalisation remains relatively limited in person-centred social service settings (Table 1).

**Table 1:** Descriptive analysis of sociodemographic, work- and health-related characteristics of the sample (*n* = 906***)***

		**N**	**%/Mean (SD)**
Age (years)			
	18–25	20	2.2
	26–35	136	15.0
	36–50	320	35.3
	51–65	426	47.0
	65 and older	4	0.4
Gender			
	Female	694	76.6
	Male	211	23.3
	Diverse	1	0.1
Experience with online counselling			
	No online counselling	13	1.4
	≤ 2 years (COVID-pandemic)	442	48.8
	3–5 years	179	19.8
	6–10 years	158	17.4
	≥ 10 years	114	12.6
Counselling field			
	General counselling	100	11.1
	Disability and mental impairment	40	4.4
	Family and parenting	183	20.2
	Children and youth	37	4.1
	Migration	69	7.6
	Debts	74	8.2
	Pregnancy	130	14.4
	Addiction	116	12.8
	Others	157	17.3
Online counselling frequency (% of an average work month)			
	Low (0–10%)	592	65.3
	Medium (20–30%)	239	26.4
	High (40% +)	75	8.3
*Working conditions*			
Autonomy			4.01 (0.61)
	Planning		3.99 (0.72)
	Methods		4.06 (0.71)
	Decision making		3.99 (0.71)
Job resources			
	Variety		4.07 (0.61)
	Collaboration (resource)		3.34 (0.84)
	Opportunities for private and work-related talks		3.64 (0.97)
	Feedback from colleagues and supervisors		3.04 (1.01)
	Social support		4.11 (0.75)
Job demands			
	Collaboration (demand)		3.45 (0.95)
	Work environment		2.21 (0.89)
	Information		2.46 (0.81)
	Workload		3.14 (0.89)
	Emotional burden (rescaled)		3.28 (0.61)
	Work interruption		2.50 (0.72)
WLB			4.31 (0.93)
*Health-related outcomes*			
General health			6.50 (2.07)
Burnout			47.88 (19.94)

A scale of 1 to 5 was used for all continuous variables except WLB (1 to 6). Health was measured on a scale from 0 to 10. Burnout was measured on a scale from 0 to 100

*Abbreviations:* *WLB* (work-life balance)

Job demands were moderate, with quantitative workload (M = 3.14, SD = 0.89) and emotional burden (M = 3.28, SD = 0.61) being particularly relevant for the HeSCare sample. Participants reported high levels of job resources, especially for variety (M = 4.07, SD = 0.61) and social support (M = 4.11, SD = 0.75). Autonomy was high in the sample, both for the total score (M = 4.01, SD = 0.61) and across the three subdimensions. While there was no indication of a significant difference in methods autonomy levels (*p* = 0.32) across online counselling frequency groups, the Kruskal–Wallis test indicated significant differences for planning autonomy (*p* = 0.02), decision-making autonomy (*p* = 0.03), and the total autonomy score (*p* = 0.03). In particular, participants in the low- and medium-user groups reported significantly lower autonomy compared with those in the high-user group. These findings show an initial tendency relevant to the competing hypotheses H1a and H1b. Overall, the observed pattern aligns more closely with the expectation that higher levels of online counselling use are associated with higher autonomy (H1a) rather than with lower autonomy (H1b). However, this pattern was not consistent across all subdimensions of autonomy. An overview of the Kruskal–Wallis test is provided in the Appendix (Additional file 1).

WLB was rated moderately high (M = 4.31, SD = 0.93). Health indicators revealed lower self-perceived health (M = 6.50, SD = 2.07) and slightly elevated burnout symptoms (M = 47.88, SD = 19.94) compared with reference values [43, 56].

Autonomy was positively correlated with job resources, particularly variety (rho = 0.40, *p* < 0.001), and negatively with job demands (Table 2). WLB showed positive associations with autonomy (rho = 0.19, *p* < 0.001) and job resources, while correlating negatively with job demands. Both autonomy and WLB correlated positively with general health (rho = 0.17 and rho = 0.39; both *p* < 0.001) and negatively with burnout (rho = −0.18 and rho = −0.49; both *p* < 0.001), suggesting that higher autonomy as well as higher WLB are associated with better health and reduced burnout. In contrast, higher job demands were associated with lower general health and higher burnout. The subdimensions of autonomy were also correlated with better general health and reduced burnout, though they displayed distinct patterns with other workplace factors. Methods and decision-making autonomy were positively associated with variety (both rho = 0.41, *p* < 0.001), whereas planning autonomy showed negative correlations with workload (rho = −0.15, *p* < 0.001) and emotional burden (rho = −0.11, *p* < 0.001).

**Table 2:** Spearman correlations of continuous variables (*n* = 906)

			**1**	**2**	**3**	**4**	**5**	**6**	**7**	**8**	**9**	**10**	**11**	**12**	**13**	**14**	**15**	**16**
1	Autonomy (summary)	rho	– –															
		p	.															
2	Autonomy (planning)	rho	0.82	– –														
		p	< 0.001^***^	.														
3	Autonomy (methods)	rho	0.85	0.52	– –													
		p	< 0.001^***^	< 0.001^***^	.													
4	Autonomy (decisions)	rho	0.88	0.58	0.68	– –												
		p	< 0.001^***^	< 0.001^***^	< 0.001^***^	.												
5	Variety	rho	0.40	0.23	0.41	0.41	– –											
		p	< 0.001^***^	< 0.001^***^	< 0.001^***^	< 0.001^***^	.											
6	Collaboration (res.)	rho	0.24	0.18	0.20	0.23	0.23	– –										
		p	< 0.001^***^	< 0.001^***^	< 0.001^***^	< 0.001^***^	< 0.001^***^	.										
7	Social support	rho	0.24	0.18	0.20	0.24	0.26	0.48	– –									
		p	< 0.001^***^	< 0.001^***^	< 0.001^***^	< 0.001^***^	< 0.001^***^	< 0.001^***^	.									
8	Collaboration (dem.)	rho	0.003	−0.01	0.002	0.02	0.14	0.39	0.16	– –								
		p	0.94	0.71	0.95	0.53	< 0.001^***^	< 0.001^***^	< 0.001^***^	.								
9	Environment	rho	−0.15	−0.07	−0.13	−0.16	−0.13	−0.16	−0.15	−0.02	– –							
		p	< 0.001^***^	0.04^*^	< 0.001^***^	< 0.001^***^	< 0.001^***^	< 0.001^***^	< 0.001^***^	0.61	.							
10	Information	rho	−0.27	−0.16	−0.21	−0.32	−0.27	−0.35	−0.46	−0.03	0.34	– –						
		p	< 0.001^***^	< 0.001^***^	< 0.001^***^	< 0.001^***^	< 0.001^***^	< 0.001^***^	< 0.001^***^	0.38	< 0.001^***^	.						
11	Workload	rho	−0.11	−0.15	−0.06	−0.08	0.06	−0.10	−0.18	0.08	0.06	0.10	– –					
		p	0.001^*^	< 0.001^***^	0.06	0.02^*^	0.07	0.003^*^	< 0.001^***^	0.03^*^	0.09	0.003^*^	.					
12	Emotional dem	rho	−0.08	−0.11	−0.02	−0.07	−0.03	−0.15	−0.17	0.03	0.12	0.16	0.14	– –				
		p	0.02*	< 0.001^***^	0.48	0.03^*^	0.33	< 0.001^***^	< 0.001^***^	0.36	< 0.001^***^	< 0.001^***^	< 0.001^***^	.				
13	Interruptions	rho	−0.13	−0.08	−0.14	−0.11	−0.08	−0.10	−0.19	0.07	0.33	0.29	0.36	0.13	– –			
		p	< 0.001^***^	0.02^*^	< 0.001^***^	< 0.001^***^	0.02^*^	0.004^*^	< 0.001^***^	0.04^*^	< 0.001^***^	< 0.001^***^	< 0.001^***^	< 0.001^***^	.			
14	WLB	rho	0.19	0.21	0.11	0.17	0.10	0.19	0.21	−0.03	−0.15	−0.21	−0.36	−0.20	−0.21	– –		
		p	< 0.001^***^	< 0.001^***^	< 0.001^***^	< 0.001^***^	0.002^*^	< 0.001^***^	< 0.001^***^	0.45	< 0.001^***^	< 0.001^***^	< 0.001^***^	< 0.001^***^	< 0.001^***^	.		
15	Health	rho	0.17	0.16	0.12	0.15	0.15	0.20	0.21	0.04	−0.10	−0.18	−0.22	−0.21	−0.15	0.39	– –	
		p	< 0.001^***^	< 0.001^***^	< 0.001^***^	< 0.001^***^	< 0.001^***^	< 0.001^***^	< 0.001^***^	0.21	0.003^*^	< 0.001^***^	< 0.001^***^	< 0.001^***^	< 0.001^***^	< 0.001^***^	.	
16	Burnout	rho	−0.18	−0.16	−0.14	−0.15	−0.13	−0.21	−0.22	−0.02	0.18	0.19	0.27	0.28	0.20	−0.49	−0.53	– –
		p	< 0.001^***^	< 0.001^***^	< 0.001^***^	< 0.001^***^	< 0.001^***^	< 0.001^***^	< 0.001^***^	0.60	< 0.001^***^	< 0.001^***^	< 0.001^***^	< 0.001^***^	< 0.001^***^	< 0.001^***^	< 0.001^***^	.

*Abbreviations*: *dem* (job demand), *res* (job resource), *WLB* (work-life balance)

Significance: *p* < 0.05^*^, *p* < 0.01^**^, *p* < 0.001^***^

### Health

In line with the theoretical assumptions of the JD-R model, multivariate linear regression analyses were conducted to examine the independent associations of autonomy with health and burnout, controlling for potential confounders (age, gender, online counselling frequency), workplace factors (job demands and resources), and WLB.

Overall, the regression model (Table 3) was statistically significant, explaining 19.6% of the variance (adjusted R^2^) in general health (F(19, 886) = 12.59, *p* < 0.001). In the adjusted model, workload (β = −0.08, *p* = 0.02), emotional burden (β = −0.11, *p* < 0.001), and WLB (β = 0.30, *p* < 0.001) were significantly associated with general health. Health outcomes did not differ by digital counselling frequency.

**Table 3:** Multiple regression model–health (n = 906)

		**B**	**SE**	**β**	**t**	***p***
Constant		3.40	1.07		3.17	0.002^*^
Online counselling frequency (Reference: high)	Low	0.33	0.24	0.08	1.40	0.16
	Medium	0.10	0.25	0.02	0.41	0.68
Autonomy		0.15	0.12	0.04	1.25	0.21
Resources	Variety	0.22	0.12	0.07	1.90	0.06
	Social support	0.03	0.10	0.01	0.31	0.76
	Collaboration (resource)	0.17	0.10	0.07	1.74	0.08
Demands	Environment	0.04	0.08	0.02	0.51	0.61
	Information	−0.06	0.10	−0.02	−0.63	0.53
	Workload	−0.17	0.08	−0.08	−2.12	0.04^*^
	Interruption	−0.11	0.10	−0.04	−1.10	0.27
	Collaboration (demand)	0.03	0.07	0.01	0.40	0.69
	Emotional burden	−0.38	0.11	−0.11	−3.54	< 0.001^***^
WLB		0.69	0.08	0.31	9.02	< 0.001^***^

Age and gender were added as control variables to the model. A range of 1 to 5 was used for all continuous variables except WLB (1 to 6). Health was measured on a scale from 0 to 10

*Abbreviations*: *WLB* (work-life balance)

While autonomy showed a significant positive correlation with health in bivariate analyses, this relationship was no longer significant in the multivariate models. Based on the results of the multivariate regression, Hypothesis H2a was not supported.

In the hierarchical regression analysis, autonomy was initially significantly associated with better health (second step); however, this association became non-significant once job resources were added (fourth step) and remained non-significant after the inclusion of WLB (final step). This pattern indicates that the relationships among autonomy, job demands, resources, WLB, and health are complex. Consistent with the JD-R model, these results suggest that autonomy may be indirectly related to health through its associations with job demands, job resources, and WLB, rather than showing a direct association. These findings provide a theoretical rationale for testing mediation.

**Fig. 1:**
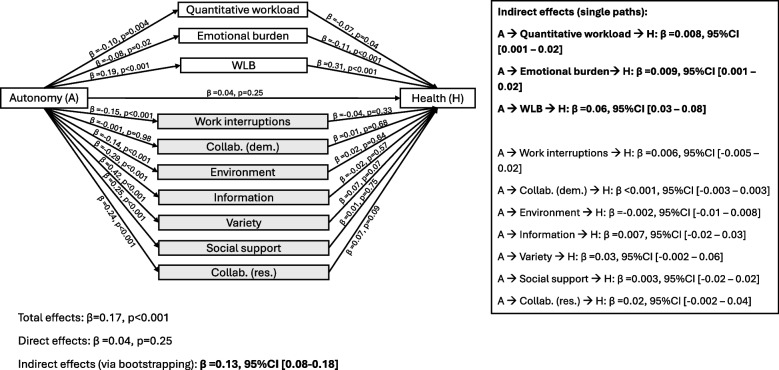
Parallel mediation analysis for the outcome health. Standardized coefficients are presented. All models are adjusted for gender and age. Indirect pathways were obtained using bootstrapping and significance was estimated using the 95% confidence Interval. Abbreviations: A (autonomy), Collab. (collaboration), dem. (job demands), H (health), res.: (job resources), WLB (work-life balance)

The mediation analysis revealed that the association between autonomy and health was fully mediated by workplace factors and WLB (Fig. 1), which supports Hypothesis 3a. Bootstrapping procedures (95%CI) identified three significant indirect pathways: higher autonomy was significantly associated with lower workload (β = −0.10, *p* = 0.004) and emotional burden (β = −0.08, *p* = 0.03), which in turn were significantly associated with better health status. The resulting indirect associations were estimated as β = 0.008 [0.001–0.02] and β = 0.009 [0.001–0.02], respectively. Additionally, WLB emerged as a significant mediator, with a standardised indirect coefficient of β = 0.06 [0.03–0.08], the highest among all indirect pathways. Indirect associations through other factors were not significantly different from zero.

**Fig. 2:**
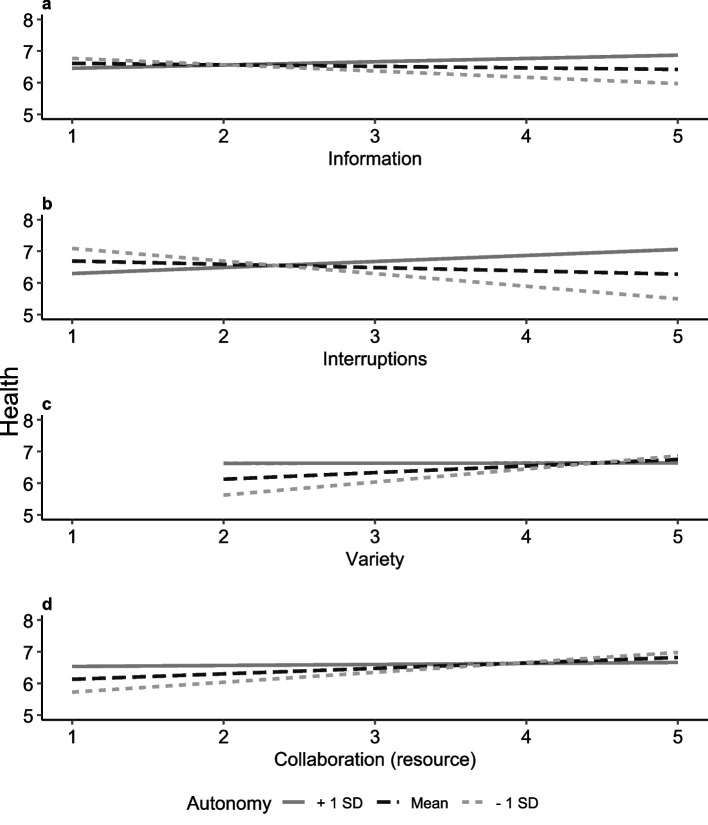
Simple slope analysis for the moderation analysis on health. Only significant interactions between predictors and autonomy are presented, including information (**a**), interruptions (**b**), variety (**c**) and collaboration (resource) (**d**). Slopes are presented for autonomy values at mean (M, black), mean minus one standard deviation (−1SD, light grey), and mean plus one standard deviation (+ 1SD, dark grey). The y-axis represents health status (scale: 0 to 10) and has been visually cropped to display values from 5 to 8

Drawing on theoretical perspectives that highlight how resources such as autonomy can shape the link between job demands and employee well-being and recognising that the availability of resources may influence how demands are appraised, we conducted a moderation analysis with autonomy as the moderating factor. Autonomy moderated the association (Table 4, Fig. 2) between interruptions and health (∆R^2^ = 1.20%, F(1, 885) = 13.81, *p* < 0.001, B(SE) = 0.22 (0.06)), with more interruptions significantly associated with lower perceived health under low-autonomy conditions (M-1SD = 3.40, B_M-1SD_ = −0.40, *p* = 0.02), but not under high-autonomy conditions (M + 1SD = 4.62, B_M+1SD_ = 0.19, *p* = 0.19). Similar patterns emerged for lack of information and involvement (∆R^2^ = 0.40%, F(1, 885) = 4.63, *p* = 0.04, B(SE) = 0.13 (0.06)), indicating a weaker negative association with health under high-autonomy conditions. In contrast, the positive association between variety and health (∆R^2^ = 0.50%, F(1, 885) = 5.62, *p* = 0.03, B(SE) = 0.13 (0.05)) was observed only under low-autonomy conditions (B_M-1SD_ = 0.41, *p* = 0.006), but not under high-autonomy conditions (B_M+1SD_ = 0.004, *p* = 0.98). Overall, these findings support Hypothesis 4a, indicating that autonomy can function as a moderator of the association between work characteristics and health.

**Table 4:** Multiple regression model–health (*n* = 906), models with significant interaction terms

		Model 1: variety x autonomy		Model 2: collaboration (res.) x autonomy		Model 3: interruption x autonomy		Model 4: information x autonomy	
		B	SE	B	SE	B	SE	B	SE
Constant		3.65^***^	1.08	3.52^*^	1.07	3.72^***^	1.07	3.62^***^	1.08
Online counselling frequency (Reference: high)	Low	0.31	0.23	0.32	0.23	0.35	0.23	0.31	0.23
	Medium	0.05	0.25	0.07	0.25	0.12	0.25	0.08	0.25
Autonomy		0.13	0.12	0.13	0.12	0.07	0.12	0.10	0.12
Resources	Variety	0.21	0.12	0.22	0.12	0.22	0.12	0.22	0.12
	Social support	0.02	0.10	0.03	0.10	0.03	0.10	0.02	0.10
	Collaboration (res.)	0.17	0.10	0.18	0.10	0.16	0.10	0.16	0.10
Demands	Environment	0.04	0.08	0.03	0.08	0.05	0.08	0.03	0.08
	Information	−0.06	0.09	−0.04	0.10	−0.08	0.09	−0.05	0.10
	Workload	−0.18^*^	0.08	−0.17^*^	0.08	−0.18^*^	0.08	−0.17^*^	0.08
	Interruption	−0.11	0.10	−0.12	0.10	−0.11	0.10	−0.12	0.10
	Collaboration (dem.)	0.03	0.07	0.03	0.07	0.03	0.07	0.03	0.07
	Emotional burden	−0.37^***^	0.11	−0.38^***^	0.11	−0.39^***^	0.11	−0.38^***^	0.11
WLB		0.69^***^	0.08	0.68^***^	0.08	0.70^***^	0.08	0.70^***^	0.08
Interaction variety X autonomy		−0.13^*^	0.05	–	–	–	–	–	–
Interaction collaboration (res.) X autonomy		–	–	−0.12^*^	0.06	–	–	–	–
Interaction interruption X autonomy		–	–	–	–	0.22^***^	0.06	–	–
Interaction information X autonomy		–	–	–	–	–	–	0.13^*^	0.06

Unstandardized coefficients (B) with standard error (SE) are reported. Variables for the interaction term were standardized before calculating the interaction term. Age and gender were added as control variables to the model. A range of 1 to 5 was used for all continuous variables except WLB (1 to 6). Health was measured on a scale from 0 to 10

*Abbreviations*: *dem* (job demand), *res* (job resource), *WLB* (work-life balance)

Significance: *p* < 0.05^*^, *p* < 0.01^**^, *p* < 0.001^***^

Despite no significant interaction between WLB and autonomy when examined as a composite construct, stratified analyses of the autonomy subdimensions showed that planning autonomy significantly moderated the association between WLB and health (ΔR^2^ = 0.50%, F(1, 883) = 5.15, *p* = 0.02, B(SE) = −0.14 (0.06)), with WLB’s benefits appearing to diminish at higher levels of planning autonomy (Additional file 1). While higher decision-making autonomy was associated with a reduced strength of the positive association between variety and health (B(SE) = −0.12 (0.05), *p* = 0.02), higher methods autonomy attenuated the negative association between information burden and health (B(SE) = 0.15 (0.06), *p* = 0.02). Across models, all autonomy dimensions weakened the negative association between interruptions and health.

### Burnout

Based on the JD-R model, autonomy is expected to be negatively associated with burnout symptoms (H2b). To examine its independent association with this outcome, we conducted a separate linear regression analysis (Table 5).

**Table 5:** Multiple regression model–burnout (*n* = 906)

			**B**	**SE**	**β**	**t**	***p***
Constant		93.03		9.42		9.88	< 0.001^***^
Online counselling frequency (Reference: high)	Low	−0.56		2.06	−0.01	−0.27	0.79
	Medium	0.87		2.21	0.02	0.39	0.69
Autonomy		−1.03		1.04	−0.03	−0.99	0.32
Resources	Variety	−2.16		1.03	−0.07	−2.10	0.04^*^
	Social support	−2.18		0.92	−0.08	−2.38	0.02^*^
	Collaboration (resource)	0.002		0.84	< 0.001	0.003	0.99
Demands	Environment	1.55		0.69	0.07	2.26	0.02^*^
	Information	−0.59		0.83	−0.02	−0.71	0.48
	Workload	2.26		0.72	0.10	3.13	0.002^*^
	Interruption	0.02		0.90	0.001	0.02	0.98
	Collaboration (demand)	−0.59		0.65	−0.03	−0.91	0.36
	Emotional burden	4.63		0.93	0.14	4.96	< 0.001^***^
WLB		−8.69		0.67	−0.40	−13.03	< 0.001^***^

Age and gender were added as control variables to the model. A range of 1 to 5 was used for all continuous variables except WLB (1 to 6). Burnout was measured on a scale from 0 to 100

*Abbreviations*: *WLB* (work-life balance)

The regression model on burnout (Table 5) was significant with a large effect size [57], explaining 33.0% of the variance (adjusted R^2^) and F(19, 886) = 24.44, *p* < 0.001. Higher workload (β = 0.10, *p* = 0.002) and emotional burden (β = 0.14, *p* < 0.001) were significantly associated with increased burnout, while greater WLB showed a strong negative association (β = −0.40, *p* < 0.001), highlighting its role as a key protective factor. Among job resources, only variety (β = −0.07, *p* = 0.04) and social support (β = −0.08, *p* = 0.02) were significantly associated with burnout, with higher resource levels linked to lower symptoms. Women reported significantly higher burnout symptoms than men (β = −0.15, *p* < 0.001), while no differences were observed for online counselling frequency.

Although autonomy was negatively correlated with burnout in bivariate analyses, it did not show a direct or independent association in the multivariate model. Accordingly, based on the adjusted regression results, Hypothesis H2b was not supported. As with general health, the hierarchical regression analysis showed that autonomy was initially associated with burnout. However, this association became non-significant once job resources were included (fourth step). This pattern aligns with the theoretical assumptions of the JD-R model and suggests that autonomy may relate to burnout indirectly through its associations with workplace and personal-life factors, supporting the rationale for examining indirect pathways.

**Fig. 3:**
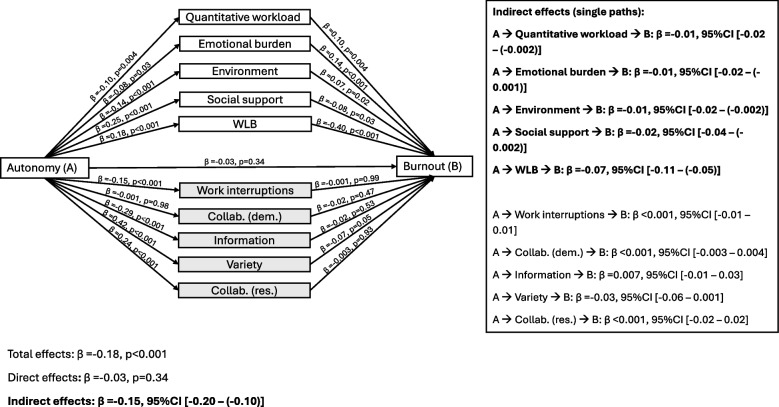
Parallel mediation analysis for the outcome burnout. Standardized coefficients are presented. All models are adjusted for gender and age. Indirect pathways were obtained using bootstrapping and significance was estimated using the 95% confidence Interval. Abbreviations: A (autonomy), B (burnout), Collab. (collaboration), dem. (job demand), res. (job resource), WLB (work-life balance)

The mediation analysis (Fig. 3) showed that autonomy’s association with burnout was fully mediated through reductions in environmental burden and quantitative workload (both β = −0.01 [−0.02 – (−0.002)]), as well as emotional burden (β = −0.01 [−0.02 – (−0.001)]). Additionally, autonomy indirectly reduced burnout by enhancing social support (β = −0.02 [−0.04 – (−0.001)]) and improving WLB (β = −0.07 [−0.11 – (−0.05)]), the strongest pathway. These findings support Hypothesis 3b.

**Fig. 4:**
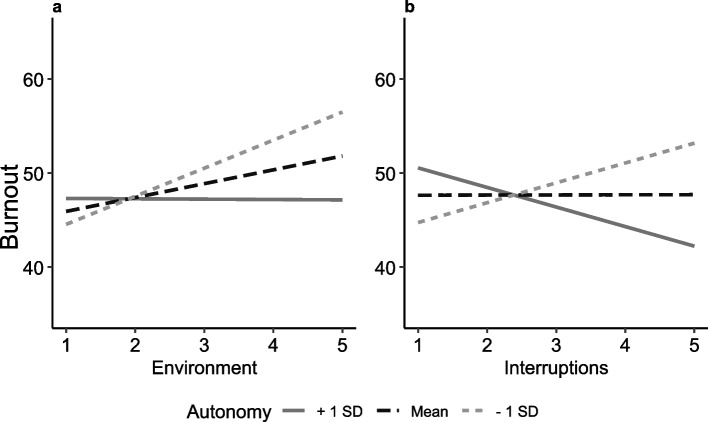
Simple slope analysis for the moderation analysis on burnout. Only significant interactions between predictors and autonomy are presented, including environment (**a**) and interruptions (**b**). Slopes are presented for autonomy values at mean (M, black), mean minus one standard deviation (−1SD, light grey), and mean plus one standard deviation (+ 1SD, dark grey). The y-axis represents burnout (scale: 0 to 100) and has been visually cropped to display values from 35 to 65

Building on theoretical perspectives suggesting that autonomy can buffer employees against the adverse effects of job demands and recognising that the availability of resources can shape how demands are appraised, we conducted a moderation analysis with autonomy specified as the moderating variable. Including interaction terms between autonomy and interruptions, and autonomy and environmental burden, significantly improved the model (∆R^2^ = 0.60%, F(1, 885) = 8.80, *p* = 0.003, B(SE) = −1.51 (0.51); and ∆R^2^ = 0.50%, F(1, 885) = 7.14, *p* = 0.008, B(SE) = −1.38 (0.52), respectively; Table 6, Fig. 4). Both demands were associated with increased burnout under low-autonomy conditions (interruptions: B_M-1SD_ = 2.11, *p* = 0.04; environmental burden: B_M-1SD_ = 2.98, *p* = 0.002), but not under high-autonomy conditions (B_M+1SD_ = −2.08, *p* = 0.08; B_M+1SD_ = −0.04, *p* = 0.97). These findings support Hypothesis 4b, indicating that autonomy can moderate the association between work characteristics and burnout, particularly for specific types of demands.

**Table 6:** Multiple regression model–burnout (*n* = 906), models with significant interaction terms

		Model 1: environment x autonomy		Model 2: interruption x autonomy	
		B	SE	B	SE
Constant		91.70^***^	9.40	90.76^***^	9.41
Online counselling frequency (Reference: high)	Low	−0.40	2.05	−0.72	2.05
	Medium	0.86	2.20	0.74	2.20
Autonomy		−0.81	1.04	−0.48	1.05
Resources	Variety	−2.05^*^	1.03	−2.15^*^	1.02
	Social support	−2.06^*^	0.91	−2.18^*^	0.91
	Collaboration (res.)	−0.04	0.83	0.05	0.83
Demands	Environment	1.48^*^	0.69	1.47^*^	0.69
	Information	−0.47	0.83	−0.47	0.83
	Workload	2.26^*^	0.72	2.27^*^	0.72
	Interruption	−0.03	0.90	−0.02	0.90
	Collaboration (dem.)	−0.69	0.65	−0.62	0.64
	Emotional burden	4.67^***^	0.93	4.70^***^	0.93
WLB		−8.70^***^	0.67	−8.76^***^	0.67
Interaction environment x autonomy		−1.38^*^	0.52	–	–
Interaction Interruption x autonomy		–	–	−1.51^*^	0.51

Unstandardized coefficients (B) with standard error (SE) are reported. Variables for the interaction term were standardized before calculating the interaction term. Age and gender were added as control variables to the model. A range of 1 to 5 was used for all continuous variables except WLB (1 to 6). Burnout was measured on a scale from 0 to 100

*Abbreviations*: *dem* (job demand), *res*. (job resource), *WLB* (work-life balance)

Significance: *p* < 0.05^*^, *p* < 0.01^**^, *p* < 0.001^***^

In stratified analyses (Additional file 1), a significant interaction was observed for planning autonomy with WLB, but not with other workplace factors. The beneficial association between WLB and burnout appeared to weaken as autonomy increased (B(SE) = 1.24 (0.54), *p* = 0.02). Methods and decision-making autonomy moderated the associations of work interruptions and environmental burden with burnout, with negative associations evident only at low levels of autonomy. Additionally, methods autonomy buffered the association between information burden and burnout, such that stronger positive associations with burnout were observed under low-autonomy conditions.

## Discussion

This study investigates the role of workplace autonomy in digital social work, an area of HeSCare, specifically examining how it functions alongside job demands and resources, WLB within the JD-R model. Rather than treating autonomy as a simple predictor, this research offers a nuanced perspective by simultaneously comparing its direct, indirect, and interactional associations with employee wellbeing. This comprehensive approach helps clarify how autonomy can function as both a resource and, in some cases, a potential stressor. This is especially relevant, as both academic work and popular discourse often portray workplace autonomy as a universal remedy for work-related stress and negative health effects. Our results demonstrate, however, that autonomy can assume distinct roles in complex digital work environments, depending upon the specific constellation of demands and resources.

Addressing our first hypothesis, we found that autonomy was high in our sample of HeSCare workers, comparable to values reported among individuals in managerial roles or self-employment [20]. While social care and therapeutic work are typically characterised by higher levels of autonomy compared with physicians and nurses [17], we observed higher total, planning, and decision-making autonomy among frequent digital counselling users compared with low- and moderate-user groups, aligning with evidence on the interplay between digital technology use and autonomy [22]. Overall, these results suggest an initial tendency more consistent with H1a than with H1b.

In line with other research, autonomy was significantly correlated with both reduced burnout symptoms and improved self-perceived health [17] in the bivariate correlation analysis. In the adjusted regression model, however, it did not retain a direct association with either outcome, resulting in the rejection of Hypotheses 2a and 2b. Mediation analysis revealed meaningful indirect pathways, particularly through WLB and specific working conditions, supporting Hypotheses 3a and 3b. This indirect effect pattern was consistent with previous research exploring pathways via enhanced creativity and work overload [58, 59]. Among all factors, work-life imbalance emerged as the strongest predictor of poor health and burnout, highlighting its central role in the psychosocial risk landscape of HeSCare work and aligning our results with previous research [10, 12]. In contrast to the traditional JD-R model, autonomy was conceptualised as a broader, contextual construct in our mediation analysis—not merely an outcome or facet of working conditions, but a factor that can actively shape them, for example through increased opportunities for job crafting [18].

Based on the traditional JD-R model, this study also aimed to explore whether autonomy operates additively or interacts with job demands to influence health outcomes in digital social care and adjacent professions, including nursing [25]. Our findings support an interactional interpretation: autonomy showed no direct associations with health or burnout, but significant moderating qualities were observed, challenging a strictly additive role of autonomy and supporting Hypotheses 4a and 4b [25]. Consistent with our findings, research indicates that, although not always predominant, interaction effects are particularly pronounced in healthcare, including social care and nursing, highlighting the beneficial role of autonomy as a potential moderator between job demands and health outcomes in these settings [25, 27]. These findings contribute to a more nuanced understanding of autonomy: whereas additive effects suggest a universal influence of autonomy, interactional effects indicate that its value is conditional, depending on specific constellations of job demands and resources [26]. On the one hand, this variability presents an opportunity to effectively identify the populations most likely to benefit from increased autonomy, similar to selective prevention settings [60]. Additionally, interaction effects can offer complementary pathways for interventions when direct changes to job demands, such as work environment issues, are not feasible due to operational, structural, or economic constraints [27]. In such cases, resources like autonomy may serve as buffers, mitigating the negative effects of high demands by providing employees with flexible strategies to tackle workplace challenges. On the other hand, this complexity challenges employers’ efforts to enhance employee well-being through workplace interventions. If applied in unsuitable conditions, increased autonomy may fail to achieve its intended goals, potentially leading to heightened stress, reduced perceptions of leadership quality, and a sense of unmet expectations [61]. In such situations, autonomy-strengthening interventions may consume valuable organisational resources that might otherwise be allocated to more effective strategies. Buffering effects may also arise because autonomy shapes how demands are appraised, aligning with the assumptions of the JD-R model [39] and suggesting that outcomes may be influenced by individual and situational evaluations of the participating employees. Interruptions, for example, emerged as a significant interaction across both health outcomes: research suggests that under high autonomy, interruptions are more likely to be appraised as manageable challenges, whereas under low autonomy, their impact follows a U-shaped curve—with both very low and very high levels associated with negative perceptions [39].

Our results also suggest that autonomy’s three subdimensions interact differently with work characteristics. Despite reductions in the detrimental associations between environmental burden and burnout under high methods and decision-making autonomy, no significant associations were found for planning autonomy. This contrasts with findings indicating that schedule flexibility may be beneficial for employee well-being [59]. However, such effects often depend on work location, with positive effects typically observed when working on-site compared with working from home, for example as part of remote counselling or telenursing. This aspect was beyond the scope of this examination.

### Theoretical and practical implications

Our findings support autonomy as a potential resource in social care and adjacent professions during the digital transformation, reinforcing its protective role in the workplace. However, its value likely depends on how well it aligns with specific job demands, resources, and workplace environments. Although individual preferences were not the focus of this study, some workers may thrive under high autonomy, whereas others may benefit more from structured supervision or collaborative practices [62]. The nuanced, indirect, and interactional associations observed support an expanded view of the JD-R model, one that considers not only interactional and additive but also mediating mechanisms (e.g., via WLB).

From a practical perspective, our findings suggest that simply increasing autonomy in social counselling roles or adjacent professions is unlikely to reduce burnout or improve health without supporting mechanisms. WLB emerged as a central mediator, indicating that autonomy is most effective when it enables employees to manage work-life boundaries. Organisations should therefore implement flexible scheduling tools and digital-disconnection practices to enhance both autonomy and WLB, while minimising the risks of boundaryless work [63]. Moreover, efforts to reduce burnout should consider how demands are appraised. Providing employees with different methods and the authority to approach and reframe demanding tasks as manageable challenges may enhance employee well-being.

Interestingly, we found no evidence of detrimental effects of autonomy on well-being as observed in previous research, in which greater autonomy, particularly in planning, was associated with increased workload and job design demands [64]. Given the high level of independent client work in social care and nursing (e.g., telenursing), our sample reported relatively high autonomy levels, comparable to those of employees in managerial positions or self-employed individuals [20]. This suggests that social counsellors may be particularly well adapted to managing autonomous work. However, our findings also indicate that the positive associations of traditional resources with well-being, such as task variety, may be attenuated under high-autonomy conditions. This could lead to inefficient use of valuable resources when interventions are implemented without a prior occupational risk assessment. Further research is needed to clarify the conditions under which autonomy may not be beneficial, including different occupational roles and work settings across various healthcare professions.

### Strengths and limitations

A key strength of this study lies in its integrated analysis of autonomy’s multiple pathways, considering additive, interactional, and mediated associations within a single framework. By focusing on a large sample of counsellors in a major German welfare organisation during a period of digital transformation, the findings provide a detailed empirical account of how autonomy functions in real life digital practice. The substantial sample size, recruited from across Germany, enhances both the statistical power of the findings and their generalizability within the national context. However, comparability to other settings may be limited due to differing occupational structures and the specific nature of digital counselling examined in this study. Employer data, nevertheless, suggests good representativeness of participants regarding gender and counselling field. Although the study was limited to social care, substantial overlap exists with other HeSCare roles, due to the wide occupational profile in social care ranging from addiction and pregnancy counselling to guidance on nursing activities in domestic care. The cross-sectional design precludes causal inference, emphasising the need for longitudinal research. Nonetheless, the findings offer valuable starting points for designing healthy work environments in complex contexts. Despite the low-threshold recruitment approach (web-link via email), self-selection bias remains a potential limitation in this study due to the use of convenience sampling and voluntary participation. Individuals with higher autonomy, for instance, may have been more inclined to participate. In addition, all data were self-reported, which may introduce reporting bias. However, the use of established and validated scales enhances the reliability of the findings. Overall, the standardised coefficients in our regression analyses were relatively small, suggesting that although several associations reached statistical significance, their practical impact may be limited. This reflects the complexity of the interaction between job demands, resources, and well-being, and suggests that autonomy alone may not be a strong predictor. Instead, autonomy likely exerts influence as part of a broader constellation of interrelated job characteristics, whose combined effects shape employee health and burnout.

## Conclusion

This study provides new insights into the complex role of autonomy in digital social counselling and adjacent fields such as nursing amid ongoing digital transformation, demonstrating that its associations with health and burnout are mainly indirect and moderated through the surrounding work context. Rather than serving as a general protective factor, autonomy’s impact appears to depend on its interaction with specific job demands and resources, such as its potential role in buffering the negative associations of frequent interruptions (*p* = 0.003) and environmental burden (*p* = 0.008) with burnout. Notably, WLB emerged as a key mediator in this relationship, with significant indirect pathways for health (β = 0.06, 95%CI [0.03–0.08]) and burnout (β = −0.07, 95%CI [−0.11- (−0.05)]). These findings expand the traditional JD-R model by highlighting the importance of both interactional and mediating processes. According to research among healthcare workers, including nurses, interactional models are more pronounced in healthcare, an observation that is consistent with our findings. While increasing autonomy holds potential in digital healthcare contexts, it is not a one-size-fits-all solution and may only be effective under certain conditions. Workplace interventions should therefore be carefully tailored to the specific setting and workforce, ensuring that autonomy supports rather than undermines employee well-being. Further longitudinal research is needed to address the limitations of our study and to better understand when and for whom autonomy is most beneficial in the evolving context of digital care work.

## Supplementary Information

Supplementary Material 1.

Supplementary Material 2.

## Data Availability

The datasets used and/or analysed during the current study are available from the corresponding author on reasonable request.
