# Supplementary material for: Decoding autonomy in digital work: how direct, indirect, and moderated effects shape health and burnout of social workers
Source: BMC Public Health. 2026 Mar 20;26:1165. doi: 10.1186/s12889-026-27041-9 (PMC13063751; doi:10.1186/s12889-026-27041-9)
Supplement: Supplementary file 1 — Supplementary Material 1. [file 12889_2026_27041_MOESM1_ESM.docx]

**Appendix/Supplementary material**

**“Decoding autonomy in digital work: How direct, indirect, and moderated effects shape health and burnout of social workers”**

Description

This appendix provides an in-depth overview of autonomy distribution across different frequencies of online counselling use. A subgroup analysis was conducted, focusing on three key dimensions of autonomy: planning autonomy, methods autonomy, and decision-making autonomy. Each autonomy dimension was examined in relation to the two outcome variables—health and burnout—using linear regression and simple slope analysis.

Table of contents

Autonomy levels by online counselling frequency 2

Confirmatory Factor Analysis 3

Health5

Table A.2: Moderated multiple regression models – health, autonomy5

Table A.3: Multiple linear regression – health, autonomy subdimensions 6

Table A.4: Moderated multiple regression models – health, planning autonomy 6

Table A.5: Moderated multiple regression models – health, methods autonomy 7

Table A.6: Moderated multiple regression models – health, decision-making autonomy8

Table A.7: Simple slope analysis – health 8

Burnout10

Table A.8: Moderated multiple regression models – burnout, autonomy 10

Table A.9: Multiple linear regression – burnout, autonomy subdimensions 11

Table A.10: Moderated multiple regression models – burnout,planning autonomy11

Table A.11: Moderated multiple regression models – burnout, methods autonomy12

Table A.12: Moderated multiple regression models – burnout, decision-making autonomy13

Table A.13: Simple slope analysis - burnout13

**Autonomy levels by online counselling frequency**

(categorized into low, medium and high users based on % of work activity per month)

A Kruskal–Wallis test was conducted as a non-parametric method to examine differences in autonomy levels across online counselling frequency groups. Frequency categories were based on Gnugesser et al.¹ and subsequently re-categorised into low (0–10%), medium (20–30%), and high users (40%+).

The test indicated a significant difference in planning autonomy across online counselling frequency per month, χ²(2, N=906)=7.70, p=0.02. Median planning autonomy scores were 4.33 for high online counselling users and 4.00 for both medium and low users. Post hoc comparisons using Dunn’s method showed that median planning autonomy scores for high-frequency users were significantly higher than those for low-frequency (p=0.01) and medium-frequency users (p=0.006).

For decision-making autonomy, the test also showed significant group differences, χ²(2, N=906)=7.07, p=0.03. However, post hoc analysis revealed a significant difference only between medium- and high-frequency users (p=0.01), whilst no significant difference was found between low- and high-frequency users (p=0.14). Median decision-making autonomy scores were 4.00 for all groups.

Significant group differences were likewise observed for total autonomy, χ²(2, N=906)=7.40, p=0.03. Post hoc analyses indicated significant differences between medium- and high-frequency users (p=0.007) and between low- and high-frequency users (p=0.05). Median total autonomy scores were 4.00 for low-frequency users, 3.89 for medium-frequency users, and 4.11 for high-frequency users.

No significant differences were found for methods autonomy, χ²(2, N=906)=2.27, p=0.32.

Reference

1. Gnugesser E, Jöllenbeck M, Schlenger W, Ochsmann E. Navigating the digital shift: Working conditions and employee health in digital social care. J Innov Knowl. 2025 May 1;10(3):100706.

**Confirmatory Factor Analysis**

| Table A.1  *Confirmatory Factor Analysis – Goodness of fit overview* | | | | |
| --- | --- | --- | --- | --- |
| Model Parameter | Model 1: Autonomy – Second-order factor analysis (based on WDQ^1^) | Model 2: Resource variables (based on KFZA^2^) | Model 3: Demand variables (based KFZA^2^ and COPSOQ^3^) | Model 4:  WLB (based on TKS^4^) |
| χ² | 149 (p<0.001) | 64 (p<0.001) | 321 (p<0.001) | 39 (p<0.001) |
| df | 24 | 17 | 39 | 5 |
| CFI | 0.99 | 0.99 | 0.97 | 0.99 |
| TLI | 0.99 | 0.99 | 0.96 | 0.99 |
| RMSEA | 0.07 (p<0.001) | 0.06 (p=0.25) | 0.09 (p<0.001) | 0.09 (p=0.008) |
| SRMR | 0.04 | 0.04 | 0.07 | 0.03 |
| *Notes.*  χ² = chi-squared; df = degrees of freedom; CFI = comparative fit index; COPSOQ = Copenhagen Psychosocial Questionnaire; KFZA = Short questionnaire for workplace analysis; RMSEA = root mean square error of approximation; SRMR = standardized root mean square residual; TKS = Trierer scale to measure work-life-balance; TLI = Tucker–Lewis Index; WDQ = Work Design Questionnaire; WLB = Work-life-balance. | | | | |

**Latent variables**

- Autonomy
  - Second order: autonomy
  - First order: Autonomy - planning, - methods, - decision making
- Demands: Environment, collaboration (demand), quantitative workload, information burden, emotional burden, interruptions
- Resources: Variety, social support, collaboration (resource)
- WLB

References

1. Stegmann S, Dick RV, Ullrich J, Charalambous J, Menzel B, Egold N, et al. Der Work Design Questionnaire: Vorstellung und erste Validierung einer deutschen Version. Z Für Arb- Organ AO. 2010 Jan;54(1):1–28.
2. Prümper J, Hartmannsgruber K, Frese M. KFZA. Kurz-Fragebogen zur Arbeitsanalyse. Z Für Arb- Organ. 1995 Jan 1;39:125–31.
3. Lincke HJ, Vomstein M, Lindner A, Nolle I, Häberle N, Haug A, et al. COPSOQ III in Germany: validation of a standard instrument to measure psychosocial factors at work. J Occup Med Toxicol Lond Engl. 2021 Nov 16;16:50.
4. Syrek C, Bauer-Emmel C, Antoni C, Klusemann J. Entwicklung und Validierung der Trierer Kurzskala zur Messung von Work-Life Balance (TKS-WLB). Diagnostica. 2011 Jul;57(3):134–45.

**Health**

**Autonomy as a composite variable**

| Table A.2  *Moderated multiple regression models – health, autonomy (n=906)* | | | | |
| --- | --- | --- | --- | --- |
| # Model | Adj. R^2^ | F (df1, df2) | ∆R^2^ (vs. #1) | p^1^ |
| 1 No interaction | 0.196 | 12.59 (19, 886) |  | <0.001^***^ |
| 2 Autonomy-interruption | 0.207 | 13.81 (1, 885) | 0.012 | <0.001^***^ |
| 3 Autonomy-collaboration (dem.) | 0.196 | 1.03 (1, 885) | 0.001 | 0.31 |
| 4 Autonomy-information | 0.199 | 4.63 (1,885) | 0.004 | 0.03* |
| 5 Autonomy-environment | 0.197 | 2.91 (1, 885) | 0.003 | 0.09 |
| 6 Autonomy-quantitative | 0.196 | 1.17 (1, 885) | 0.001 | 0.28 |
| 7 Autonomy-emotional | 0.198 | 3.14 (1, 885) | 0.003 | 0.08 |
| 8 Autonomy-social support | 0.195 | 0.36 (1, 885) | <0.001 | 0.55 |
| 9 Autonomy-variety | 0.200 | 5.62 (1, 885) | 0.005 | 0.02^*^ |
| 10 Autonomy-collaboration (res.) | 0.199 | 4.53 (1,885) | 0.004 | 0.03^*^ |
| 11 Autonomy - WLB | 0.198 | 3.03 (1, 885) | 0.003 | 0.08 |
| *Notes.* ^1^: Significant changes of the model by adding interactions terms. Abbreviations: dem. (job demand); res. (job resource); WLB (Work-life balance). | | | | |

**Autonomy subdimensions**

| **Table A.3**  ***Multiple linear regression – health, autonomy subdimensions (n=906)*** | | | | | | | |
| --- | --- | --- | --- | --- | --- | --- | --- |
|  | | | B | SE | β | T | p |
| Constant | | | 3.42 | 1.08 |  | 3.18 | 0.002^*^ |
| Online counselling frequency (Reference: High) | Low |  | 0.33 | 0.24 | 0.08 | 1.40 | 0.16 |
|  | Medium |  | 0.10 | 0.25 | 0.02 | 0.41 | 0.69 |
| Autonomy | Planning | | 0.03 | 0.11 | 0.009 | 0.23 | 0.82 |
|  | Methods | | 0.08 | 0.13 | 0.03 | 0.64 | 0.52 |
|  | Decisions | | 0.04 | 0.14 | 0.01 | 0.29 | 0.77 |
| Resources | Variety | | 0.22 | 0.12 | 0.06 | 1.80 | 0.07 |
|  | Social support | | 0.03 | 0.10 | 0.01 | 0.30 | 0.76 |
|  | Collaboration (resource) | | 0.17 | 0.10 | 0.07 | 1.73 | 0.08 |
| Demands | Enviroment | | 0.04 | 0.08 | 0.02 | 0.51 | 0.61 |
|  | Information | | -0.06 | 0.10 | -0.02 | -0.62 | 0.54 |
|  | Workload | | -0.17 | 0.08 | -0.08 | -2.12 | 0.03^*^ |
|  | Interruption | | -0.11 | 0.10 | -0.04 | -1.07 | 0.29 |
|  | Collaboration (demand) | | 0.03 | 0.07 | 0.01 | 0.41 | 0.69 |
|  | Emotional burden | | -0.38 | 0.11 | -0.11 | -3.55 | <0.001^***^ |
| WLB | | | 0.69 | 0.08 | 0.31 | 8.99 | <0.001^***^ |
| *Notes.*  All models were adjusted for age and gender. A range of 1 to 5 was employed for all continuous variables except WLB (1 – 6). Health was measured on a scale from 0 to 10.  Abbreviations: WLB (work-life balance). | | | | | | | |

| **Table A.4**  ***Moderated multiple regression models – health, planning autonomy (n=906)*** | | | | |
| --- | --- | --- | --- | --- |
| Model | Adj. R^2^ | F (df1, df2) | ∆R^2^ (vs. #1) | p^1^ |
| 1 No interaction | 0.194 | 11.57 (21, 884) |  | <0.001^***^ |
| 2 Autonomy-interruption | 0.201 | 8.73 (1, 883) | 0.008 | 0.003^*^ |
| 3 Autonomy-collaboration (dem.) | 0.193 | 0.23 (1, 883) | <0.001 | 0.63 |
| 4 Autonomy-information | 0.194 | 0.97 (1,883) | 0.001 | 0.33 |
| 5 Autonomy-environment | 0.195 | 2.04 (1, 883) | 0.002 | 0.15 |
| 6 Autonomy-quantitative / workload | 0.194 | 0.60 (1, 883) | 0.001 | 0.44 |
| 7 Autonomy-emotional | 0.195 | 2.29 (1, 883) | 0.002 | 0.13 |
| 8 Autonomy-social support | 0.193 | 0.02 (1,883) | <0.001 | 0.90 |
| 9 Autonomy-variety | 0.196 | 2.82 (1, 883) | 0.003 | 0.09 |
| 10 Autonomy-collaboration (res.) | 0.196 | 2.00 (1, 883) | 0.002 | 0.10 |
| 11 Autonomy - WLB | 0.198 | 5.15 (1, 883) | 0.005 | 0.02^*^ |
| *Notes.* ^1^: Significant changes of the model by adding interactions terms. All models were adjusted for age, gender, online counselling frequency and the autonomy aspects methods and decision making. Abbreviations: dem. (job demands), res. (job resource), WLB (work-life balance). | | | | |

| **Table A.5**  ***Moderated multiple regression models – health, methods autonomy (n=906)*** | | | | |
| --- | --- | --- | --- | --- |
| Model | Adj. R^2^ | F (df1, df2) | ∆R^2^ (vs. #1) | p^1^ |
| 1 No interaction | 0.194 | 11.57 (21, 884) |  | <0.001^***^ |
| 2 Autonomy-interruption | 0.202 | 9.96 (1, 883) | 0.009 | 0.002^*^ |
| 3 Autonomy-collaboration (dem.) | 0.195 | 2.36 (1, 883) | 0.002 | 0.13 |
| 4 Autonomy-information | 0.199 | 6.88 (1, 883) | 0.006 | 0.009^*^ |
| 5 Autonomy-environment | 0.194 | 1.29 (1, 883) | 0.001 | 0.26 |
| 6 Autonomy-quantitative / workload | 0.194 | 1.15 (1, 883) | 0.001 | 0.28 |
| 7 Autonomy-emotional | 0.195 | 2.08 (1, 883) | 0.002 | 0.15 |
| 8 Autonomy-social support | 0.193 | 0.41 (1, 883) | <0.001 | 0.52 |
| 9 Autonomy-variety | 0.201 | 9.25 (1, 883) | 0.008 | 0.002^*^ |
| 10 Autonomy-collaboration (res.) | 0.197 | 4.10 (1, 883) | 0.004 | 0.04^*^ |
| 11 Autonomy - WLB | 0.194 | 0.95 (1, 883) | 0.001 | 0.33 |
| *Notes.* ^1^: Significant changes of the model by adding interactions terms. All models were adjusted for age, gender, online counselling frequency and the autonomy aspects planning and decision making. Abbreviations: dem. (demand), res. (resource), WLB (work-life balance). | | | | |

| **Table A.6**  ***Moderated multiple regression models – health, decision-making autonomy (n=906)*** | | | | |
| --- | --- | --- | --- | --- |
| Model | Adj. R^2^ | F (df1, df2) | ∆R^2^ (vs. #1) | p^1^ |
| 1 No interaction | 0.194 | 11.57 (21, 884) |  | <0.001^***^ |
| 2 Autonomy-interruption | 0.204 | 12.60 (1, 883) | 0.01 | <0.001^***^ |
| 3 Autonomy-collaboration (dem.) | 0.194 | 0.45 (1, 883) | <0.001 | 0.50 |
| 4 Autonomy-information | 0.196 | 3.48 (1, 883) | 0.003 | 0.06 |
| 5 Autonomy-environment | 0.196 | 3.30 (1, 8853) | 0.003 | 0.07 |
| 6 Autonomy-quantitative / workload | 0.194 | 0.78 (1, 883) | 0.001 | 0.38 |
| 7 Autonomy-emotional | 0.195 | 2.60 (1, 883) | 0.002 | 0.11 |
| 8 Autonomy-social support | 0.194 | 0.52 (1, 883) | <0.001 | 0.47 |
| 9 Autonomy-variety | 0.198 | 565 (1, 883) | 0.005 | 0.02^*^ |
| 10 Autonomy-collaboration (res.) | 0.197 | 3.93 (1, 883) | 0.003 | 0.048^*^ |
| 11 Autonomy - WLB | 0.195 | 1.76 (1, 883) | 0.002 | 0.19 |
| *Notes.* ^1^: Significant changes of the model by adding interactions terms. All models were adjusted for age, gender, online counselling frequency and the autonomy aspects methods and planning. Abbreviations: dem. (job demands), res. (job resource), WLB (work-life balance). | | | | |

| **Table A.7**  ***Simple slope analysis – health, autonomy subdimensions (n=906)*** | | | | | |
| --- | --- | --- | --- | --- | --- |
| Interaction term | B (SE)^1^ | Simple slope analysis^2^ | | | |
|  |  | Autonomy value | | B | p |
| Autonomy planning x Interruptions | 0.18^**^ (0.06) | M-1SD | 3.27 | -0.36 | 0.04^*^ |
|  |  | M | 3.99 | -0.11 | 0.33 |
|  |  | M+1SD | 4.70 | 0.14 | 0.32 |
| Autonomy planning x WLB | -0.14^*^ (0.06) | M-1SD | 3.27 | 0.84 | <0.001^***^ |
|  |  | M | 3.99 | 0.69 | <0.001^***^ |
|  |  | M+1SD | 4.70 | 0.54 | <0.001^***^ |
| Autonomy methods x Information | 0.15^**^ (0.06) | M-1SD | 3.35 | -0.24 | 0.07 |
|  |  | M | 4.06 | -0.06 | 0.61 |
|  |  | M+1SD | 4.76 | 0.13 | 0.33 |
| Autonomy methods x Interruptions | 0.18^**^ (0.06) | M-1SD | 3.35 | -0.35 | 0.03^*^ |
|  |  | M | 4.06 | -0.10 | 0.36 |
|  |  | M+1SD | 4.76 | 0.14 | 0.32 |
| Autonomy methods x Variety | -0.16^**^ (0.05) | M-1SD | 3.35 | 0.46 | 0.004^*^ |
|  |  | M | 4.06 | 0.20 | 0.12 |
|  |  | M+1SD | 4.76 | -0.06 | 0.69 |
| Autonomy methods x Collaboration (res.) | -0.12^*^ (0.06) | M-1SD | 3.35 | 0.31 | 0.01^*^ |
|  |  | M | 4.06 | 0.17 | 0.07 |
|  |  | M+1SD | 4.76 | 0.03 | 0.79 |
| Autonomy decision-making x Interruptions | 0.21^***^ (0.06) | M-1SD | 3.29 | -0.40 | 0.02^*^ |
|  |  | M | 3.99 | -0.10 | 0.36 |
|  |  | M+1SD | 4.70 | 0.19 | 0.20 |
| Autonomy decision-making x Variety | -0.12^*^ (0.05) | M-1SD | 3.29 | 0.41 | 0.007^*^ |
|  |  | M | 3.99 | 0.21 | 0.10 |
|  |  | M+1SD | 4.70 | 0.005 | 0.98 |
| Autonomy decision-making x Collaboration (res.) | -0.11^*^ (0.06) | M-1SD | 3.29 | 0.31 | 0.01^*^ |
|  |  | M | 3.99 | 0.17 | 0.07 |
|  |  | M+1SD | 4.70 | 0.04 | 0.71 |
| *Notes.*  ^1^: Unstandardised regression coefficient (B), SE, and significance level for the interaction term in the health regression model. Each interaction was tested in a separate model, controlling for age, gender, online counselling frequency, job demands, job resources, work–life balance, and all three autonomy dimensions (planning, methods, and decision-making). Health was measured on a scale from 0-10.  ^2^: Unstandardised regression coefficient (B) and significance level for the predictor at M–1SD, M, and M+1SD of the autonomy variable. All continuous variables ranged from 1–5, except work–life balance, which ranged from 1–6.  Abbreviations: WLB (Work-life balance), res. (job resource).  Significance: p<0.05*, p<0.01**, p<0.001***. | | | | | |

**Burnout**

**Autonomy as a composite variable**

| Table A.8  *Moderated multiple regression models – burnout, autonomy (n=906)* | | | | |
| --- | --- | --- | --- | --- |
| # Model | Adj. R^2^ | F (df1, df2) | ∆R^2^ (vs. #1) | p^1^ |
| 1 No interaction | 0.330 | 24.44 (19, 886) |  | <0.001^***^ |
| 2 Autonomy-interruption | 0.336 | 8.80 (1, 885) | 0.006 | 0.003^*^ |
| 3 Autonomy-collaboration (dem.) | 0.329 | 0.007 (1, 885) | <0.001 | 0.93 |
| 4 Autonomy-information | 0.330 | 1.98 (1, 885) | 0.001 | 0.16 |
| 5 Autonomy-environment | 0.334 | 7.14 (1, 885) | 0.005 | 0.008^*^ |
| 6 Autonomy-quantitative | 0.331 | 2.45 (1, 885) | 0.002 | 0.12 |
| 7 Autonomy-emotional | 0.329 | 0.31 (1, 885) | <0.001 | 0.58 |
| 8 Autonomy-social support | 0.330 | 1.90 (1, 885) | 0.001 | 0.17 |
| 9 Autonomy-variety | 0.330 | 0.95 (1, 885) | 0.001 | 0.33 |
| 10 Autonomy-collaboration (res.) | 0.331 | 2.13 (1, 885) | 0.002 | 0.15 |
| 11 Autonomy – WLB | 0.330 | 1.68 (1, 885) | 0.001 | 0.20 |
| *Notes.*  ^1^: Significant changes of the model by adding interactions terms. Abbreviations: dem. (job demands); res. (job resource); WLB (Work-life balance). | | | | |

**Autonomy subdimensions**

| **Table A.9**  ***Multiple linear regression – burnout, autonomy subdimensions (n=906)*** | | | | | | | |
| --- | --- | --- | --- | --- | --- | --- | --- |
|  | | | B | SE | β | t | p |
| Constant | | | 92.24 | 9.45 |  | 9.77 | <0.001^***^ |
| Online counselling frequency (Reference: High) | Low |  | -0.54 | 2.06 | -0.01 | -0.26 | 0.79 |
|  | Medium |  | 0.93 | 2.21 | 0.02 | 0.42 | 0.67 |
| Autonomy | Planning | | 0.31 | 0.98 | 0.01 | 0.31 | 0.76 |
|  | Methods | | -1.70 | 1.13 | -0.06 | -1.51 | 0.13 |
|  | Decisions | | 0.32 | 1.21 | 0.01 | 0.27 | 0.79 |
| Resources | Variety | | -1.95 | 1.05 | -0.06 | -1.86 | 0.06 |
|  | Social support | | -2.16 | 0.92 | -0.08 | -2.36 | 0.02^*^ |
|  | Collaboration (resource) | | 0.01 | 0.84 | <0.001 | 0.01 | 0.99 |
| Demands | Enviroment | | 1.57 | 0.69 | 0.07 | 2.27 | 0.02^*^ |
|  | Information | | -0.55 | 0.84 | -0.02 | -0.65 | 0.52 |
|  | Workload | | 2.27 | 0.72 | 0.10 | 3.15 | 0.002^*^ |
|  | Interruption | | -0.11 | 0.91 | -0.004 | -0.12 | 0.91 |
|  | Collaboration (demand) | | -0.60 | 0.65 | -0.03 | -0.93 | 0.35 |
|  | Emotional burden | | 4.70 | 0.94 | 0.14 | 5.02 | <0.001^***^ |
| WLB | | | -8.77 | 0.67 | -0.41 | -13.07 | <0.001^***^ |
| *Notes.*  All models were adjusted for age and gender. A range of 1 to 5 was employed for all continuous variables except WLB (1 – 6). Burnout was measured on a scale from 0 to 100.  Abbreviations: WLB (work-life balance). | | | | | | | |

| **Table A.10**  ***Moderated multiple regression models – burnout, planning autonomy (n=906)*** | | | | |
| --- | --- | --- | --- | --- |
| Model | Adj. R^2^ | F (df1, df2) | ∆R^2^ (vs. #1) | p^1^ |
| 1 No interaction | 0.330 | 22.18 (21, 884) |  | <0.001^***^ |
| 2 Autonomy-interruption | 0.330 | 2.19 (1, 883) | 0.002 | 0.14 |
| 3 Autonomy-collaboration (dem.) | 0.330 | 0.99 (1, 883) | 0.001 | 0.32 |
| 4 Autonomy-information | 0.329 | 0.14 (1, 883) | <0.001 | 0.71 |
| 5 Autonomy-environment | 0.329 | 0.37 (1, 883) | <0.001 | 0.54 |
| 6 Autonomy-quantitative / workload | 0.330 | 2.16 (1, 883) | 0.002 | 0.14 |
| 7 Autonomy-emotional | 0.329 | 0.13 (1, 883) | <0.001 | 0.72 |
| 8 Autonomy-social support | 0.331 | 2.36 (1, 883) | 0.002 | 0.13 |
| 9 Autonomy-variety | 0.329 | 0.29 (1, 883) | <0.001 | 0.59 |
| 10 Autonomy-collaboration (res.) | 0.331 | 3.56 (1, 883) | 0.003 | 0.06 |
| 11 Autonomy - WLB | 0.333 | 5.21 (1, 883) | 0.004 | 0.02^*^ |
| *Notes.* ^1^: Significant changes of the model by adding interactions terms. Abbreviations: dem. (job demands), res. (job resource), WLB (work-life balance). | | | | |

| **Table A.11**  ***Moderated multiple regression models – burnout, methods autonomy (n=906)*** | | | | | |
| --- | --- | --- | --- | --- | --- |
| Model | Adj. R^2^ | | F (df1, df2) | ∆R^2^ (vs. #1) | p^1^ |
| 1 No interaction | | 0.330 | 22.18 (21, 884) |  | <0.001^***^ |
| 2 Autonomy-interruption | | 0.337 | 10.99 (1, 883) | 0.008 | <0.001^***^ |
| 3 Autonomy-collaboration (dem.) | | 0.329 | 0.004 (1, 883) | <0.001 | 0.95 |
| 4 Autonomy-information | | 0.332 | 4.75 (1, 883) | 0.004 | 0.03^*^ |
| 5 Autonomy-environment | | 0.338 | 12.18 (1, 883) | 0.009 | <0.001^***^ |
| 6 Autonomy-quantitative / workload | | 0.329 | 0.83 (1, 883) | 0.001 | 0.36 |
| 7 Autonomy-emotional | | 0.329 | 0.34 (1, 883) | <0.001 | 0.56 |
| 8 Autonomy-social support | | 0.331 | 2.40 (1, 883) | 0.002 | 0.12 |
| 9 Autonomy-variety | | 0.332 | 3.67 (1, 883) | 0.003 | 0.06 |
| 10 Autonomy-collaboration (res.) | | 0.330 | 1.77 (1, 883) | 0.001 | 0.16 |
| 11 Autonomy – WLB | | 0.329 | 0.005 (1, 883) | <0.001 | 0.94 |
| *Notes.* ^1^: Significant changes of the model by adding interactions terms. Abbreviations: dem. (job demands), res. (job resource), WLB (work-life balance). | | | | | |
|  | |  |  |  |  |

| **Table A.12**  ***Moderated multiple regression models – burnout, decision-making autonomy (n=906)*** | | | | |
| --- | --- | --- | --- | --- |
| Model | Adj. R^2^ | F (df1, df2) | ∆R^2^ (vs. #1) | p^1^ |
| 1 No interaction | 0.330 | 22.18 (21, 884) |  | <0.001^***^ |
| 2 Autonomy-interruption | 0.334 | 7.19 (1, 883) | 0.005 | 0.007^*^ |
| 3 Autonomy-collaboration (demand) | 0.329 | 0.61 (1, 883) | <0.001 | 0.43 |
| 4 Autonomy-information | 0.329 | 0.78 (1, 883) | 0.001 | 0.38 |
| 5 Autonomy-environment | 0.335 | 7.80 (1, 883) | 0.006 | 0.005^*^ |
| 6 Autonomy-quantitative / workload | 0.330 | 2.29 (1, 883) | 0.002 | 0.13 |
| 7 Autonomy-emotional | 0.329 | 0.23 (1, 883) | <0.001 | 0.63 |
| 8 Autonomy-social support | 0.329 | 0.20 (1, 883) | <0.001 | 0.65 |
| 9 Autonomy-variety | 0.330 | 1.54 (1, 883) | 0.001 | 0.22 |
| 10 Autonomy-collaboration (resource) | 0.329 | 0.45 (1, 883) | <0.001 | 0.50 |
| 11 Autonomy - WLB | 0.330 | 1.04 (1, 883) | 0.001 | 0.31 |
| *Notes.* ^1^: Significant changes of the model by adding interactions terms. Abbreviations: dem. (job demands), res. (job resource), WLB (work-life balance). | | | | |

| **Table A.13**  ***Simple slope analysis – burnout, autonomy subdimensions (n=906)*** | | | | | |
| --- | --- | --- | --- | --- | --- |
| Interaction term | β (SE)^1^ | Simple slope analysis | | | |
|  |  | Autonomy value | | B^2^ | p |
| Autonomy planning x WLB | 1.24^*^ (0.54) | M-1SD | 3.27 | -9.85 | <0.001^***^ |
|  |  | M | 3.99 | -8.70 | <0.001^***^ |
|  |  | M+1SD | 4.70 | -7.56 | <0.001^***^ |
| Autonomy methods x Environment | -1.84^***^ (0.53) | M-1SD | 3.35 | 3.55 | <0.001^***^ |
|  |  | M | 4.06 | 1.53 | 0.02^*^ |
|  |  | M+1SD | 4.76 | -0.49 | 0.59 |
| Autonomy methods x Information | -1.11^*^ (0.51) | M-1SD | 3.35 | 0.73 | 0.49 |
|  |  | M | 4.06 | -0.60 | 0.53 |
|  |  | M+1SD | 4.76 | -1.94 | 0.11 |
| Autonomy methods x Interruption | -1.65^***^ (0.50) | M-1SD | 3.35 | 2.16 | 0.04^*^ |
|  |  | M | 4.06 | -0.11 | 0.90 |
|  |  | M+1SD | 4.76 | -2.39 | 0.04^*^ |
| Autonomy decision-making x Environment | -1.39^**^ (0.50) | M-1SD | 3.29 | 3.00 | <0.001^***^ |
|  |  | M | 3.99 | 1.46 | 0.03^*^ |
|  |  | M+1SD | 4.70 | -0.09 | 0.93 |
| Autonomy decision-making x Interruption | -1.42^**^ (0.53) | M-1SD | 3.29 | 1.86 | 0.08 |
|  |  | M | 3.99 | -0.10 | 0.91 |
|  |  | M+1SD | 4.70 | -2.07 | 0.09 |
| *Notes.*  ^1^: Unstandardised regression coefficient (B), SE, and significance level for the interaction term in the burnout regression model. Each interaction was tested in a separate model, controlling for age, gender, online counselling frequency, job demands, job resources, work–life balance, and all three autonomy dimensions (planning, methods, and decision-making). Burnout was measured on a scale from 0-100.  ^2^: Unstandardised regression coefficient (B) and significance level for the predictor at M–1SD, M, and M+1SD of the autonomy variable. All continuous variables ranged from 1–5, except work–life balance, which ranged from 1–6.  Abbreviations: WLB (Work-life balance), res. (job resource).  Significance: p<0.05^*^, p<0.01^**^, p<0.001^***^. | | | | | |
