# Supplementary material for: Decoding autonomy in digital work: how direct, indirect, and moderated effects shape health and burnout of social workers
Source: BMC Public Health. 2026 Mar 20;26:1165. doi: 10.1186/s12889-026-27041-9 (PMC13063751; doi:10.1186/s12889-026-27041-9)
Supplement: Supplementary file 2 — Supplementary Material 2. [file 12889_2026_27041_MOESM2_ESM.pdf]

Appendix/Supplementary Material

Questionnaire

Description

Survey instrument used in the study: “Decoding autonomy in digital work: How direct, indirect, and moderated effects shape health and burnout of social workers”

It includes the original German items used for data collection and an English translation provided for transparency purposes.

Befragung in Kooperation zwischen dem Institut für Arbeitsmedizin der Universität zu Lübeck und

der Berufsgenossenschaft für Gesundheitsdienst und Wohlfahrtspflege (BGW)

gemeinsam mit dem Deutschen Caritasverband (DCV)

Digitale Transformation bei der Caritas

DiCarO - Den digitalen Wandel in der Caritas-Online-Beratung gesund gestalten

Sehr geehrte Online-Beratende der Caritas,

Digitalisierung verändert unsere Arbeit: die Arbeitsorganisation, die Arbeitsinhalte, das soziale Miteinander. Dies gilt zunehmend auch für Beratung und Beratende der Caritas. Um diesen Prozess gesundheitsfördernd zu gestalten, wurde die Studie DiCarO ins Leben gerufen.

Worauf zielt die Studie ab?

Mit dem vorliegenden Online-Fragebogen für DiCarO soll zunächst ein "Status Quo" Ihrer digitalen Arbeitssituation erhoben werden, um wichtige Handlungsfelder für Verbesserungen zu identifizieren und Zusammenhänge zwischen Digitalisierung und Ihrer Gesundheit bzw. Ihrem Wohlbefinden aufzudecken. Letztendlich soll so eine Organisationsentwicklung für Online-Beratung angestoßen werden.

Wie lange dauert die Befragung?

Die Bearbeitung des Fragebogens wird durch Ihre Antworten gesteuert und nimmt ungefähr 30 – 40 Minuten in Anspruch.

Warum ist Ihre Teilnahme so wichtig?

Wir wissen natürlich, dass Ihr Arbeitstag voller Termine ist und 30 - 40 min sehr viel Zeit sind. Wir wissen aber auch, dass nur Sie Ihren Arbeitsalltag wirklich gut einschätzen und beschreiben können und dass vor allem Sie Ideen zu guten Lösungsansätzen für problematische Bereiche haben.

Daher möchten wir Sie herzlich einladen die Befragung zu nutzen, Ihr persönliches Erleben anonym darzustellen. Natürlich sind die Antwortmöglichkeiten bei standardisierten Fragebögen begrenzt. Daher haben wir uns bemüht, auch einige Freitext-Möglichkeiten in den Fragebogen einzubauen. Zusätzlich versprechen wir Ihnen weit gefächerte Fragen, die vielleicht dazu anregen, den eigenen Arbeitsalltag und das eigene Wohlbefinden einmal kritisch zu beleuchten.

Vielen Dank dafür, dass Sie sich diese Zeit für unseren Fragebogen und Ihre Arbeitssituation nehmen!

Was wird gefragt?

Es geht um Sie. Wir möchten Ihre Arbeitssituation umfassend beleuchten und fragen deshalb sehr gründlich ab, z.B. zu

- Ihren Arbeitsinhalten
- Ihrer Arbeitsorganisation
- der Arbeitsumgebung
- Zusammenarbeit mit anderen
- Arbeitszufriedenheit
- spezifische Belastungen und Beanspruchungen
- Funktionalität der Beratungs-Software.

Auch ein paar gezielte Fragen zu Ihrer Gesundheit/Ihrem Wohlbefinden sind dabei.

Wir bitten Sie, den nachfolgenden Fragebogen spontan und ohne langes Nachdenken zu bearbeiten.

Wie sind die Möglichkeiten zur Teilnahme und der Datenschutz geregelt?

Die Teilnahme an dieser Studie ist selbstverständlich freiwillig und vollständig anonym. Sie starten die Auswertung, indem Sie den versendeten Link nutzen. Auswertungen werden nur auf Gruppenbasis von mindestens 7 Personen durchgeführt.

Um die Arbeitssituation im zeitlichen Verlauf bewerten zu können, möchten wir in ca. 1-1,5 Jahren eine Nach-Befragung durchführen. Deshalb bitten wir Sie im Fragebogen um die Vergabe eines Pseudonyms. Das heißt: Wir bitten Sie zu Beginn der Befragung, freiwillig ein persönliches Codewort zu vergeben.

Dies ist einfach und nur Sie kennen den Code. Dazu geben wir im Fragebogen eine konkrete Anleitung. Der Code wird nur zum Zusammenführen der zeitversetzten Datensätze genutzt und taucht natürlich sonst nirgendwo auf.

Die Ergebnisse der Befragung und weitere Informationen zum Projekt DiCarO werden Ihnen selbstverständlich im weiteren Studienverlauf vorgestellt. Dazu werden Sie nach Abschluss der Befragung gesondert informiert.

Vielen Dank für Ihr Interesse und Ihre Teilnahme!

Sollten Sie weitere Informationen über die Studie wünschen, kontaktieren Sie gerne die

Studienkoordinatorin.

[Kontaktdetails entfernt]

Anmerkungen zum Datenschutz:

Die von Ihnen eingegebenen Daten (Rohdaten) werden auf dem deutschen Server des Online-Befragungsinstruments (www.soscisurvey.de) ohne Erfassung der IP-Adressen gesammelt. Der Befragungsserver stellt eine Datenschutz-Konformität nach DSGVO und BDSG sowie eine durchgehende SSL-verschlüsselte Datenübertragung sicher. Eine Rückverfolgung der Angaben zu einzelnen Personen ist somit vollkommen ausgeschlossen.

Ich habe die schriftliche Information zur oben genannten Studie erhalten, gelesen und verstanden. Ich wurde darin über das Ziel und den Verlauf der Studie und die Freiwilligkeit der Teilnahme aufgeklärt. Ich erkläre hiermit meine Teilnahme an der oben genannten Studie. Meine Teilnahme ist freiwillig und mir ist bekannt, dass ich das Recht habe, diese jederzeit ohne Angabe von Gründen zu beenden, ohne dass mir dadurch Nachteile entstehen. Ich willige in die pseudonyme Erhebung, Verarbeitung, Speicherung meiner Daten ein. Ich willige ein, dass die erhobenen Daten anonym und auf Gruppenbasis (Diözese/Beratungsbereich, Gruppengrößen von mind. 7 Personen) ausgewertet und zurückgemeldet und ggf. zur Interventionsplanung herangezogen werden.

- Ja
- Nein

Soziodemographische Daten und Arbeitssituation

Wir stellen zunächst ein paar Fragen zu Ihrer Person und Ihrer Arbeitssituation, um die Gruppe der Teilnehmenden besser einordnen zu können.

Welches Geschlecht haben Sie?

o Weiblich o Männlich o Divers o keine Angabe

Welcher Altersgruppe gehören Sie an?

- o Unter 18 Jahren
- o 18 bis 25 Jahren
- o 26 bis 35 Jahre
- o 36 bis 50 Jahre
- o 51 bis 65 Jahre
- o Älter als 65 Jahre
- o keine Angabe

In welchem Beratungsfeld sind Sie hauptsächlich/überwiegend tätig?

- o Allgemeine Sozialberatung
- o Behinderung und psychische Beeinträchtigung
- o Beratung zur Pflege Angehöriger
- o Eltern und Familie
- o HIV und Aids
- o Hospiz- und Palliativberatung
- o Kinder und Jugendliche
- o Kinder- und Jugend-Reha
- o Krisenberatung für junge Erwachsene
- o Kuren für Mütter und Väter
- o Lebensberatung/Eheberatung
- o Leben im Alter
- o Migration
- o Opferbetreuung
- o Psychotherapie (Beratungspsychologie)
- o Psychotherapie (klinische Therapie)

- rechtliche Betreuung und Vorsorge
- Schulden
- Schuldnerberatung für junge Leute
- Schwangerschaft
- Straffälligkeit
- Sucht
- Trauerberatung
- U25 Suizidprävention
- Übergang von Schule zu Beruf
- Sonstiges (Bitte nachfolgend Freitext einfügen)

Seit wann führen Sie Onlineberatungen durch?

- seit ca. 2 Jahren oder kürzer (ungefährer Beginn mit Coronapandemie 03/2020 oder später)
- seit 3 bis 5 Jahren
- seit 6 bis 10 Jahren
- länger als 10 Jahre
- keine Angabe
- Ich führe keine Onlineberatungen durch.

Wenn Sie Ihren typischen Arbeitsalltag betrachten: Welchen prozentualen Anteil nehmen Onlineberatungen pro Monat ein? (Schätzung in 10% Schritten)

- Ausschließlich Onlineberatung (100%)
- 90%
- 80%
- 70%
- 60%
- 50%
- 40%
- 30%
- 20%
- 10%
- Keine Onlineberatung (0%)

Arbeitsinhalt und Organisation

Die nächsten Fragen beziehen sich auf klassische Aspekte der Arbeit (z. B. Inhalte, Organisation, Umgebungsfaktoren). Zunächst möchten wir mehr zu Ihren Arbeitsinhalten und Ihrem Arbeitsalltag erfahren.

Die folgenden Fragen betreffen die Planbarkeit Ihres Arbeitsalltags. Inwiefern treffen die folgenden Aussagen auf ihre Arbeitstätigkeit zu?

Diese Aussage trifft zu…

	Überhaupt nicht (1)	Eher nicht (2)	Teils/ teils (3)	Eher (4)	Völlig (5)	Keine Angabe
Ich bin frei in der zeitlichen Einteilung meiner Arbeit.	o	o	o	o	o	o
Ich kann selbst entscheiden, in welcher Reihenfolge ich meine Arbeit mache.	o	o	o	o	o	o
Ich kann meine Arbeit so planen, wie ich es möchte.	o	o	o	o	o	o
Meine Arbeit ermöglicht es mir, Initiative zu übernehmen und nach eigenem Ermessen zu handeln.	o	o	o	o	o	o
Ich kann bei meiner Arbeit viele Entscheidungen selbständig treffen.	o	o	o	o	o	o
Meine Arbeit gewährt mir einen großen Entscheidungsspielraum.	o	o	o	o	o	o
Bei meiner Arbeit kann ich oft zwischen verschiedenen Herangehensweisen wählen.	o	o	o	o	o	o
Ich kann selbst entscheiden, mit welchen Mitteln ich zum Ziel komme.	o	o	o	o	o	o
Ich habe viele Freiheiten in der Art und Weise, wie ich meine Arbeit verrichte.	o	o	o	o	o	o

Diese Aussage trifft zu…

	Gar nicht (1)	Wenig (2)	Mittelmäßig (3)	Überwiegend (4)	Völlig (5)	Keine Angabe
Ich kann bei meiner Arbeit Neues dazulernen.	o	o	o	o	o	o

Ich kann mein Wissen und Können bei meiner Arbeit voll einsetzen.	o	o	o	o	o	o
Bei meiner Arbeit habe ich, insgesamt gesehen, häufig wechselnde, unterschiedliche Arbeitsaufgaben.	o	o	o	o	o	o

Arbeitsumgebung und Information

Bitte bewerten Sie Ihre Arbeitsumgebung sowie die Ihnen zur Verfügung gestellten Arbeitsmittel und Informationen.

Diese Aussage trifft zu…

	Gar nicht (1)	Wenig (2)	Mittelmäßig (3)	Überwiegend (4)	Völlig (5)	Keine Angabe
Oft stehen mir die benötigten Informationen, Materialien und Arbeitsmittel (z. B. Computer) nicht zur Verfügung.	o	o	o	o	o	o
An meinem Arbeitsplatz gibt es ungünstige Umgebungsbedingungen, wie Lärm, Klima, Staub.	o	o	o	o	o	o
An meinem Arbeitsplatz sind Räume und Raumausstattung ungenügend (ergonomische Möbel, Lichtverhältnisse, Belüftungssysteme etc.) ausgestattet.	o	o	o	o	o	o

Wie schätzen Sie die Weitergabe wichtiger Informationen bei Ihrer Dienststelle ein?

Diese Aussage trifft zu…

	In sehr hohem Maße (1)	In hohem Maße (2)	Zum Teil (3)	In geringem Maße (4)	In sehr geringem Maße (5)	Keine Angabe
Werden Sie rechtzeitig im Voraus über Veränderungen an Ihrem Arbeitsplatz informiert, z. B. über wichtige Entscheidungen,	o	o	o	o	o	o

Veränderungen oder Pläne für die Zukunft?						
Erhalten Sie alle Informationen, die Sie brauchen, um Ihre Arbeit gut zu erledigen?	o	o	o	o	o	o

Arbeitsanforderungen und soziale Rückendeckung

Wie erleben Sie folgende Anforderungen bei Ihrer Arbeit?

In welchem Maß stimmen Sie den folgenden Aussagen zu Ihren Belastungen bei der Arbeit zu?

	Gar nicht (1)	Wenig (2)	Mittelmäßig (3)	Überwiegend (4)	Völlig (5)	Keine Angabe
Ich werde bei meiner eigentlichen Arbeit immer wieder unterbrochen (z. B. durch das Telefon).	o	o	o	o	o	o
Ich habe zu viel Arbeit.	o	o	o	o	o	o
Häufig stehe ich unter Zeitdruck.	o	o	o	o	o	o
Ich fühle mich von der Menge an Informationen, die ich täglich verarbeiten muss, überwältigt.	o	o	o	o	o	o

Wie erleben Sie die emotionalen Anforderungen Ihrer Arbeit?

Diese Aussage trifft zu…

	Immer (1)	Oft (2)	Manchmal (3)	Selten (4)	Nie/ fast nie (5)	Keine Angabe
Ist Ihre Arbeit emotional fordernd?	o	o	o	o	o	o
Verlangt Ihre Arbeit von Ihnen, dass Sie Ihre Gefühle verbergen?	o	o	o	o	o	o
Verlangt Ihre Arbeit von Ihnen, sich mit Ihrer Meinung zurückzuhalten?	o	o	o	o	o	o

Wie erleben Sie den sozialen Rückhalt an Ihrem Arbeitsplatz?

Wie gestaltet sich das soziale Miteinander in Ihrem Arbeitsalltag?

Diese Aussage trifft zu…

	Gar nicht (1)	Wenig (2)	Mittelmäßig (3)	Überwiegend (4)	Völlig (5)	Keine Angabe	Trifft nicht zu
Meine Arbeit erfordert enge Zusammenarbeit mit anderen Personen in der Abteilung.	o	o	o	o	o	o	o
Ich kann mich während der Arbeit mit verschiedenen Kolleg*innen über dienstliche und private Dinge unterhalten.	o	o	o	o	o	o	o
Ich bekomme von Vorgesetzten und Kolleg*innen immer Rückmeldung über die Qualität meiner Arbeit.	o	o	o	o	o	o	o
Ich kann mich auf meine Kolleg*innen verlassen, wenn es schwierig wird.	o	o	o	o	o	o	o
Ich kann mich auf meine(n) direkte(n) Vorgesetzten verlassen, wenn es bei der Arbeit schwierig wird.	o	o	o	o	o	o	o
Man hält in der Abteilung gut zusammen.	o	o	o	o	o	o	o

Work-Life Balance

Gerade bei digitaler Arbeit wird die Grenze zwischen Arbeit und Freizeit fließender. Ungeklärt ist die Frage, ob Mitarbeiter mit digitalen Arbeitsinhalten sich noch ausreichend erholen können und wie digitale Arbeit gestaltet werden muss, um eine nach wie vor gute Erholung von Beschäftigten zu ermöglichen. Dazu möchten wir Ihnen ein paar Fragen stellen.

Bitte geben Sie an, inwieweit Sie den folgenden Aussagen zustimmen.

	Stimme gar nicht zu (1)	Stimme überwiegend nicht zu (2)	Stimme eher nicht zu (3)	Stimme eher zu (4)	Stimme überwiegend zu (5)	Stimme völlig zu (6)	Keine Angabe
Ich bin zufrieden mit meiner Balance zwischen Arbeit und Privatleben.	o	o	o	o	o	o	o
Es fällt mir schwer, Berufs- und Privatleben miteinander zu vereinbaren.	o	o	o	o	o	o	o
Ich kann die Anforderungen aus meinem Privatleben und die Anforderungen aus meinem Berufsleben gleichermaßen gut erfüllen.	o	o	o	o	o	o	o
Es gelingt mir, einen guten Ausgleich zwischen belastenden und erholsamen Tätigkeiten in meinem Leben zu erreichen.	o	o	o	o	o	o	o
Ich bin damit zufrieden, wie meine Prioritäten in Bezug auf den Beruf und das Privatleben verteilt sind.	o	o	o	o	o	o	o

Wie bewerten Sie Ihren derzeitigen Gesundheitszustand?

Skala von 0 (schlechtester Gesundheitszustand) bis 10 (bester Gesundheitszustand)

0	1	2	3	4	5	6	7	8	9	10
o	o	o	o	o	o	o	o	o	o	o

Energie und psychisches Wohlbefinden:

Bitte geben Sie für jede der folgenden Aussagen an, inwieweit sie für Sie zutrifft.

Wie häufig...

	Immer (100)	Oft (75)	Manchmal (50)	Selten (25)	Nie/ fast nie (0)	Keine Angabe
... sind Sie körperlich erschöpft?	o	o	o	o	o	o
... sind Sie emotional erschöpft?	o	o	o	o	o	o
... fühlen Sie sich ausgelaugt?	o	o	o	o	o	o
... kommen Sie zur Arbeit, obwohl Sie sich richtig krank und unwohl fühlen?	o	o	o	o	o	o

Herzlichen Dank für Ihre Teilnahme!

Weitere Fragen? Sehr gerne! Melden Sie sich einfach per E-Mail.

Mit besten Grüßen, das Forschungsteam der Universität zu Lübeck

ENGLISH VERSION

(only for transparency purposes; not used in the data collection process)

Survey in cooperation between the Institute of Occupational Medicine, Prevention and Workplace Health Management at the University of Lübeck and the Employer's Liability Insurance Association for Health Services and Welfare Care (BGW), together with the German Caritas Association (DCV)

Digital Transformation at Caritas

DiCarO – Healthily designing the digital shift in Caritas online counseling

Dear Caritas Online Counsellors,

Digitalization is changing our work: work organization, work content, and social interaction. This increasingly applies to counselling and counsellors at Caritas. To design this process in a health-promoting manner, the DiCarO study was initiated.

What is the aim of the study?

The purpose of this online questionnaire for DiCarO is to first record a "status quo" of your digital work situation in order to identify important fields of action for improvements and to uncover correlations between digitalization and your health or well-being. Ultimately, this is intended to initiate organizational development for online counseling.

How long does the survey take?

The process of the questionnaire is guided by your answers and takes approximately 30–40 minutes.

Why is your participation so important?

We know, of course, that your workday is full of appointments and that 30–40 minutes is a lot of time. However, we also know that only you can truly assess and describe your daily work routine and that, above all, you have ideas for good solutions in problematic areas.

Therefore, we would like to cordially invite you to use this survey to present your personal experiences anonymously. Naturally, the response options in standardized questionnaires are limited. Therefore, we have endeavored to include some free-text options in the

questionnaire. Additionally, we promise you a wide range of questions that may encourage you to critically examine your own daily work routine and well-being.

Thank you for taking this time for our questionnaire and your work situation!

What will be asked?

It is about you. We want to shed light on your work situation comprehensively and therefore ask very thoroughly, e.g., about:

- Your work content
- Your work organization
- The work environment
- Cooperation with others
- Job satisfaction
- Specific stressors and strains
- Functionality of the counselling software.

A few targeted questions about your health/well-being are also included.

We ask you to complete the following questionnaire spontaneously and without long deliberation.

How are the options for participation and data protection regulated?

Participation in this study is, of course, voluntary and completely anonymous. You start the evaluation by using the Email web link. Evaluations are only carried out on a group basis of at least 7 people.

In order to be able to evaluate the work situation over time, we would like to conduct a follow-up survey in approx. 1–1.5 years. Therefore, we ask you to assign a pseudonym in the questionnaire. This means: At the beginning of the survey, we ask you to voluntarily assign a personal code word. This is simple, and only you know the code. We provide specific instructions for this in the questionnaire. The code is only used to merge the time-delayed data sets and, of course, does not appear anywhere else.

The results of the survey and further information on the DiCarO project will, of course, be presented to you as the study progresses. You will be informed separately about this after the survey has been completed. Thank you for your interest and your participation! Should you require further information about the study, please feel free to contact the Study Coordinator.

[Contact details removed for anonymity]

Notes on Data Protection:

The data you enter (raw data) is collected on the German server of the online survey instrument (www.soscisurvey.de) without recording IP addresses. The survey server ensures data protection compliance in accordance with the GDPR (DSGVO) and the Federal Data Protection Act (BDSG), as well as continuous SSL-encrypted data transmission. Any tracing of information back to an individual person is therefore completely excluded.

Informed Consent:

I have received, read, and understood the written information regarding the above-mentioned study. I have been informed about the aim and course of the study and the voluntary nature of participation. I hereby declare my participation in the above-mentioned study. My participation is voluntary, and I am aware that I have the right to terminate it at any time without providing reasons and without incurring any disadvantages. I consent to the pseudonymized collection, processing, and storage of my data. I consent to the collected data being evaluated and reported back anonymously and on a group basis (diocese/counseling area, group sizes of at least 7 people) and, if necessary, being used for intervention planning.

o Yes

o No

Socio-Demographic Data and Work Situation

We will begin with a few questions about yourself and your work situation to help us categorize the group of participants.

What is your gender?

o Female o Male o Diverse o Prefer not to say

To which age group do you belong?

o Under 18 years

o 18 to 25 years

o 26 to 35 years

o 36 to 50 years

o 51 to 65 years

o Older than 65 years

o Prefer not to say

In which field of counseling are you mainly/predominantly active?

o General social counseling

o Disability and mental impairment

o Counseling for caregivers of relatives

o Parents and family

o HIV and AIDS

o Hospice and palliative care counseling

o Children and adolescents

o Child and youth rehabilitation

o Crisis counseling for young adults

o Retreats for mothers and fathers

o Life counseling/Marriage counseling

o Living in old age

o Migration

o Victim support

o Psychotherapy (Counseling psychology)

o Psychotherapy (Clinical therapy)

o Legal guardianship and advocacy

o Debt

o Debt counseling for young people

o Pregnancy

o Delinquency

o Addiction

o Bereavement counseling

o U25 Suicide prevention

o Transition from school to work

o Other (Please specify): __________

How long have you been conducting online counseling?

o Approx. 2 years or less (Started around the beginning of the COVID-19 pandemic 03/2020 or later)

o 3 to 5 years

o 6 to 10 years

o Longer than 10 years

o Prefer not to say

o I do not conduct online counseling.

Considering your typical daily work routine: What percentage per month is taken up by online counseling? (Estimated in 10% increments)

o Exclusively online counseling (100%)

o 90%

…[increments 80% to 20%] ...

o 10%

o No online counseling (0%)

Work Content and Organization

The next questions refer to classic aspects of work (e.g., content, organization, environmental factors). First, we would like to learn more about your work content and your daily work routine.

The following questions concern the predictability of your daily work routine. To what extent do the following statements apply to your work activity?

	Not at all (1)	Rather not (2)	Partly (3)	Rather (4)	Completely (5)	Prefer not to say
I am free to arrange my own working hours.	o	o	o	o	o	o
I can decide for myself in which order I do my work.	o	o	o	o	o	o
I can plan my work as I wish.	o	o	o	o	o	o
My work allows me to take initiative and act at my own discretion.	o	o	o	o	o	o
I can make many decisions independently in my work.	o	o	o	o	o	o
My work grants me a high degree of decision-making latitude.	o	o	o	o	o	o
In my work, I can often choose between different approaches.	o	o	o	o	o	o
I can decide for myself which means I use to achieve my goal.	o	o	o	o	o	o
I have a lot of freedom in the way I perform my work.	o	o	o	o	o	o

	Not at all (1)	A little (2)	Moderately (3)	Predominantly (4)	Completely (5)	Prefer not to say
I can learn new things in my work.	o	o	o	o	o	o
I can fully apply my knowledge and skills in my work.	o	o	o	o	o	o
Overall, I have frequently changing, diverse tasks in my work.	o	o	o	o	o	o

Work Environment and Information

Please rate your work environment and the tools and information provided to you.

	Not at all (1)	A little (2)	Moderately (3)	Predominantly (4)	Completely (5)	Prefer not to say
Often, the required information, materials, and equipment are not available to me.	o	o	o	o	o	o
There are unfavorable environmental conditions at my workplace (noise, climate, dust).	o	o	o	o	o	o
The rooms and room equipment at my workplace are inadequate (ergonomic furniture, etc.).	o	o	o	o	o	o

How do you assess the transfer of important information at your office?

	To a very high degree (1)	To a high degree (2)	In part (3)	To a low degree (4)	To a very low degree (5)	Prefer not to say
Are you informed in good time about changes at your workplace (e.g., important decisions, changes, or future plans)?	o	o	o	o	o	o
Do you receive all the information you need to do your job well?	o	o	o	o	o	o

Job Demands and Social Support

To what extent do you agree with the following statements regarding your stressors at work?

	Not at all (1)	A little (2)	Moderately (3)	Predominantly (4)	Completely (5)	Prefer not to say
I am repeatedly interrupted during my actual work (e.g., by the telephone).	o	o	o	o	o	o
I have too much work.	o	o	o	o	o	o
I am often under time pressure.	o	o	o	o	o	o
I feel overwhelmed by the amount of information I have to process daily.	o	o	o	o	o	o

How do you experience the emotional demands of your work?

	Always (1)	Often (2)	Sometimes (3)	Seldom (4)	Never / Almost never (5)	Prefer not to say
Is your work emotionally demanding?	o	o	o	o	o	o

Does your work require you to hide your feelings?	o	o	o	o	o	o
Does your work require you to hold back your opinion?	o	o	o	o	o	o

How do you experience social support at your workplace?

How is the social interaction in your daily work routine?

	Not at all (1)	A little (2)	Moderately (3)	Predominantly (4)	Completely (5)	Prefer not to say	Does not apply
My work requires close cooperation with others in the department.	o	o	o	o	o	o	o
I can talk to colleagues about work-related and private matters.	o	o	o	o	o	o	o
I receive feedback from supervisors and colleagues on my work quality.	o	o	o	o	o	o	o
I can rely on my colleagues when things get difficult.	o	o	o	o	o	o	o
I can rely on my direct supervisor when things get difficult at work.	o	o	o	o	o	o	o
There is good cohesion in the department.	o	o	o	o	o	o	o

Work-Life Balance

In the context of digital work in particular, the boundaries between work and private life are becoming increasingly blurred. The question remains as to whether employees with digital work content are still able to recover sufficiently, and how digital work must be designed to

ensure that employees can continue to recover effectively. We would like to ask you a few questions regarding this topic.

Please indicate the extent to which you agree with the following statements.

	Do not agree at all (1)	Mostly disagree (2)	Somewhat disagree (3)	Somewhat agree (4)	Mostly agree (5)	Completely agree (6)	Prefer not to say
I am satisfied with the balance between my work and private life.	o	o	o	o	o	o	o
It is difficult for me to reconcile professional and private life.	o	o	o	o	o	o	o
I can fulfill the demands of my private life and professional life equally well.	o	o	o	o	o	o	o
I manage a good balance between stressful and relaxing activities.	o	o	o	o	o	o	o
I am satisfied with how my priorities regarding work/private life are distributed.	o	o	o	o	o	o	o

Health Status

How do you rate your current state of health?

(Scale from 0 = worst health status to 10 = best health status)

**0**	**1**	**2**	**3**	**4**	**5**	**6**	**7**	**8**	**9**	**10**
o	o	o	o	o	o	o	o	o	o	o

Energy and Psychological Well-being

How often...	Always (100)	Often (75)	Sometimes (50)	Seldom (25)	Never / Almost never (0)	Prefer not to say
... are you physically exhausted?	o	o	o	o	o	o
... are you emotionally exhausted?	o	o	o	o	o	o
... do you feel worn out?	o	o	o	o	o	o
... do you come to work even though you feel really sick and unwell?	o	o	o	o	o	o

Thank you very much for your participation!

Any further questions? We are happy to help! Simply contact us by e-mail.

Best regards, the Research Team at the University of Lübeck.
